# Unique Terminal Regions and Specific Deletions of the Segmented Double-Stranded RNA Genome of Alternaria Alternata Virus 1, in the Proposed Family Alternaviridae

**DOI:** 10.3389/fmicb.2021.773062

**Published:** 2021-10-22

**Authors:** Chien-Fu Wu, Nanako Aoki, Naoki Takeshita, Toshiyuki Fukuhara, Hiroshi X. Chiura, Tsutomu Arie, Ioly Kotta-Loizou, Ryo Okada, Ken Komatsu, Hiromitsu Moriyama

**Affiliations:** ^1^Laboratory of Molecular and Cellular Biology, Tokyo University of Agriculture and Technology, Fuchu, Japan; ^2^Laboratory of Plant Pathology, Tokyo University of Agriculture and Technology, Fuchu, Japan; ^3^Department of Life Sciences, Faculty of Natural Sciences, Imperial College London, London, United Kingdom

**Keywords:** mycovirus, dsRNA virus, *Alternaria alternata*, 5' cap structure, viral protein, Alternaviridae, deletion of RNA genome

## Abstract

Alternaria alternata virus 1 (AaV1) has been identified in the saprophytic fungus *Alternaria alternata* strain EGS 35–193. AaV1 has four genomic double-stranded (ds)RNA segments (dsRNA1–4) packaged in isometric particles. The 3' end of each coding strand is polyadenylated (36–50nt), but the presence of a cap structure at each 5' end has not previously been investigated. Here, we have characterized the AaV1 genome and found that it has unique features among the mycoviruses. We confirmed the existence of cap structures on the 5' ends of the AaV1 genomic dsRNAs using RNA dot blots with anti-cap antibodies and the oligo-capping method. Polyclonal antibodies against purified AaV1 particles specifically bound to an 82kDa protein, suggesting that this protein is the major capsid component. Subsequent Edman degradation indicated that the AaV1 dsRNA3 segment encodes the major coat protein. Two kinds of defective AaV1 dsRNA2, which is 2,794bp (844 aa) in length when intact, appeared in EGS 35–193 during subculturing, as confirmed by RT-PCR and northern hybridization. Sequence analysis revealed that one of the two defective dsRNA2s contained a 231bp deletion, while the other carried both the 231bp deletion and an additional 465bp deletion in the open reading frame. Both deletions occurred in-frame, resulting in predicted proteins of 767 aa and 612 aa. The fungal isolates carrying virions with the defective dsRNA2s showed impaired growth and abnormal pigmentation. To our best knowledge, AaV1 is the first dsRNA virus to be identified with both 5' cap and 3'poly(A) structures on its genomic segments, as well as the specific deletions of dsRNA2.

## Introduction

Mycoviruses are viruses that infect fungi and are ubiquitous in most fungal genera (Ghabrial et al., 2015). The first mycovirus to be identified was found in cultivated mushrooms in 1962 (Hollings, 1962). Since then, the numbers of reports on mycoviruses have been increasing (Ghabrial and Suzuki, 2009; Ghabrial et al., 2015). Viruses related to mycoviruses have been isolated from insects (Koyama et al., 2015; Liu et al., 2016), plants (Li et al., 2009; Nibert et al., 2014), and other lower eukaryotic organisms, such as oomycetes (Hacker et al., 2005; Cai et al., 2019; Uchida et al., 2021). According to the International Committee on Taxonomy of Viruses, most mycoviruses have linear double-stranded (ds) RNA genomes (e.g., *Chrysoviridae*, *Reoviridae*, *and Totiviridae*), linear positive/negative single-stranded (+/−ss) RNA genomes (e.g., *Alphaflexiviridae* and *Hypoviridae*), or reverse transcribing (RT) ssRNA genomes (*Metaviridae and Pseudoviridae*). One family of circular ssDNA mycoviruses, *Genomoviridae*, has been reported to date, while new families are being proposed to accommodate novel mycoviruses. Most mycoviruses have no 3' poly(A) tails or 5' cap structures, although members of *Alphaflexiviridae* and *Pseudoviridae* have both 5' cap structures and 3' poly(A) tails (Lefkowitz et al., 2017). Members of *Metaviridae* and *Hypoviridae* have genome segments with only the 3' poly(A) tails (Li et al., 2015; Lefkowitz et al., 2017), whereas *Reoviridae* viruses have only the 5' caps and no 3' poly(A) tails (Furuichi et al., 1976).

Many mycoviruses are latent, but a growing number of reports indicate that they can modulate traits of their host fungi (Ghabrial et al., 2015; Kotta-Loizou and Coutts, 2017). The well-known mycovirus Cryphonectria parasitica hypovirus 1, which infects the chestnut blight fungus *Cryphonectria parasitica*, reduces pigmentation and sporulation and attenuates host virulence (McCabe and Van Alfen, 2002; Nuss, 2005). In the white root rot fungus *Rosellinia necatrix*, at least five mycovirus families have been discovered and used to study virus-host and virus-virus interactions (Kondo et al., 2013). Infection by Magnaporthe oryzae chrysovirus 1-D changes the host’s morphology and causes abnormal pigmentation by decreasing accumulation of the melanin biosynthesis intermediate scytalone (Higashiura et al., 2019). Alternaria alternata chrysovirus 1 infection downregulates host growth due to viral RNA accumulation and upregulates host virulence by increasing the production of AK-toxin during spore germination (Okada et al., 2018). A unique ssDNA mycovirus, Sclerotinia sclerotiorum hypovirulence-associated DNA virus, causes hypovirulence in its plant pathogenic host fungus and has potential as a biological control agent (Yu et al., 2010). Rearrangements of dsRNA genomes, spontaneous extension, or deletion events can be frequently observed in the major genera of Reoviridae (Desselberger, 1996). Additionally, the inducible genomes rearrangement was also reported in mycoreovirus (Sun and Suzuki, 2008; Eusebio-Cope et al., 2010; Kanematsu et al., 2014).

A new viral family, Alternaviridae, was proposed in 2013 (Kozlakidis et al., 2013) and currently accommodates seven species, including Alternaria alternata virus 1 (AaV1; Table 1). AaV1 was the first alternavirus to be completely sequenced. It has four genomic dsRNA segments (dsRNA1–4; 3.6–1.4 kbp in size), packaged in an isometric virion about 33nm in diameter. The dsRNA1 encodes a protein with the conserved motifs of an RNA-dependent RNA polymerase (RdRp); however, the glycine residue is replaced by an alanine in the most conserved GDD motif (Aoki et al., 2009; Moriyama et al., 2021). A property of AaV1 is the presence of intact poly(A) tails (36 to 50nt) at the 3' terminal regions of all four dsRNA molecules. The presence of capping structures at the 5' ends has not previously been investigated (Aoki et al., 2009). AaV1 infection leads to phenotypic alterations in the saprophytic fungus *Alternaria alternata* (strain EGS 35–193), including irregular pigmentation, decreased mycelial growth, collapsed aerial hyphae, and cytolysis in the hyphae (Aoki et al., 2009).

**Table 1:** List of current members in the proposed family Alternaviridae.

Virus	Genome	Particle size	5' cap	3' poly(A) tail	Reference
Alternaria alternata virus 1 (AaV1)	dsRNA1 (3,617nt, RdRp)	33nm	+	+	Aoki et al., 2009
	dsRNA2 (2,794nt, P2)				
	dsRNA3 (2,576nt, Coat protein)				
	dsRNA4 (1,420nt, P4)				
Aspergillus mycovirus 341 (AsV341)	dsRNA1 (3,588nt, RdRp)	nd	nd	+	Hammond et al., 2008
Aspergillus foetidus mycovirus (AfV-F)	dsRNA1 (3,588nt, RdRp)	nd	nd	+	Kozlakidis et al., 2013
	dsRNA2 (2,770nt, P2)				
	dsRNA3 (2,466nt, P3)				
	dsRNA4 (2005nt, P4)				
Fusarium poae alternavirus 1 (FpAV1)	dsRNA1 (3,559nt, RdRp)	nd	nd	+	Osaki et al., 2016
	dsRNA2 (2,496nt, P2)				
	dsRNA3 (2,482nt, P3)				
Fusarium graminearum alternavirus 1 (FgAV1)	dsRNA1 (3,524nt, RdRp)	nd	nd	+	He et al., 2018
	dsRNA2 (2,470nt, P2)				
	dsRNA3 (2,485nt, P3)				
Fusarium incarnatum alternavirus 1 (FiAV1)	dsRNA1 (3,548nt, RdRp)	nd	nd	+	Zhang et al., 2019
	dsRNA2 (2,514nt, P2)				
	dsRNA3 (2,498nt, P3)				
Aspergillus heteromorphus alternavirus 1 (AheAV1)	dsRNA1 (3,576nt, RdRp)	nd	nd	+	Gilbert et al., 2019
	dsRNA2 (2,742nt, P2)				
	dsRNA3 (2,427nt, P3)				
nd: no data					

Here, we show that AaV1 has a 7-methylguanosine (m^7^G)-cap structure on the 5' end of each dsRNA segment. Additionally, spontaneous in-frame deletions have been detected in the AaV1 genome, and these may be related to the impaired growth of the fungal host.

## Materials and Methods

### Fungal Isolates and Culture Conditions

The AaV1-infected *A. alternata* isolate EGS 35–193 was described previously (Johnson et al., 2001; Aoki et al., 2009). We realized that the original isolate EGS 35–193 contained not only an intact dsRNA2 segment but a dsRNA2 segment with an in-frame deletion. Therefore, we named the original EGS 35–193 as EGS 35–193-1d. During the subculturing of EGS 35–193-1d on YGA plates (0.5% yeast extract, 2% glucose, and 1% agar), we found two other isolates, EGS 35–193-0d and EGS 35–193-2d. EGS 35–193-0d has only the intact dsRNA2, while EGS 35–193-2d has the intact dsRNA2 and two other kinds of dsRNA2 segments with either one or two internal deletions. All isolates were cultured on YGA plates, PDA plates (200g/l unpeeled potato slices, 20g/l dextrose, and 15g/l agar), and V8A plates (200ml/lV8 juice, 3g/l CaCO_3_, 15g/l agar) at 25°C for one week. For liquid cultures, mycelial plugs were used to inoculate YG broth (0.5% yeast extract and 2% glucose) and grown at 25°C for two weeks with shaking (60 strokes per min).

### Curing of an AaV1-Infected *A. alternata* Isolate

The isolate EGS 35–193 was cured of AaV1 infection using a modified fragmentation treatment (Supplementary Figures S1A and S1B; Kim et al., 2013). Mycelia were collected after one week of incubation on YGA plates, suspended in autoclaved distilled water, chilled on ice for 40s, and then fragmented twice for 5s each time using a tissue homogenizer (Precellys^®^24., Bertin Corp, MD, United States) set at 5000rpm. After chilling on ice for a further 10s, the fragmented mycelia were spread on YGA plates and cultured for 1–2days at 25°C and then in YG broth for two weeks at 25°C. The process was repeated until no AaV1 was detectable.

### Purification of Virus Particles and Antibody Production

We purified AaV1 virions as described previously (Aoki et al., 2009), with minor modifications. The entire process was carried out at 4°C. Briefly, 10g (fresh weight) of mycelia was homogenized in 100ml buffer (0.1M sodium phosphate, 0.2M KCl, pH 7.4) with a mixer and a French Press (One Shot A Model, Constant Systems, U.K.; 35Kpsi). Next, the homogenate was mixed for 1h with 40% (v/v) chloroform/n-butanol (1:1), and the mixture was centrifuged at 8,000*g* (TOMY Suprema 21, NA-8 rotor, Japan) for 20min. The supernatant was adjusted to 8% (w/v) polyethylene glycol 6,000 and 1% (w/v) NaCl and gently stirred for 3h to overnight. The solution was then centrifuged at 10,000*g* (TOMY Suprema 21, NA-8 rotor) for 5min, and the pellet was resuspended in 8ml 0.05M sodium phosphate buffer (pH 7.0) and left at 4°C overnight. Finally, the suspension was layered onto 15ml of a 45% sucrose cushion and centrifuged at 69,260g (Hitachi CP80WX, P28S swing rotor) for 16h at 4°C, and the pellet was resuspended in 0.05M sodium phosphate buffer (pH 7.0).

We also purified Saccharomyces cerevisiae virus L-A (ScV-L-A) virus particles from *Saccharomyces cerevisiae* strain YPH499 and Mycoreovirus 1 (MyRV1) virus particles from chestnut blight fungus using the methods described by Powilleit et al. (2007) and Hillman et al. (2004), respectively.

The purified virus particles were negatively stained with 2% uranyl acetate and then observed by transmission electron microscope (JEM 1400 Plus, JOEL, Japan) with an acceleration voltage of 80kV.

For anti-AaV1 antiserum production, we obtained about 1.5mg of partially purified AaV1 proteins from 100g (fresh weight) of EGS 35–193-1d grown in YG broth (Aoki et al., 2009). These purified proteins were injected into rabbits (about 0.2mg per injection) every week for 3weeks, and then, the rabbits were given further injections (about 0.05mg per injection) every week for another 3weeks to ensure the success of the immunization (Protein Purify, Isesaki, Japan). The antiserum was collected after the fifth injection over a period of three weeks. The immunoglobulin G (IgG) against AaV1 was then purified from the anti-AaV1 antiserum using protein A agarose (Funakoshi, Japan) and then stored at-80°C.

### Protein Analysis

Purified virus particles were analyzed by 8% SDS-PAGE and either stained with Coomassie Brilliant Blue (CBB; EzStainAQua, ATTO, Japan) or transferred to PVDF membrane (ATTO, Japan) for western blotting assays. For the western assays, the proteins were first exposed to the anti-AaV1 primary antiserum (1:5000 dilution) and then to a secondary HRP-conjugated goat anti-rabbit polyclonal antibody (Bio-Rad; 1:10000 dilution). After washing, antibody-bound proteins were detected by luminescence using the EzWestLumi plus and EZ-Capture MG system (ATTO, Japan).

We also isolated the major 82kDa viral protein for sequence analysis. Purified AaV1 virus particles (50μg protein) were resolved by 8% SDS-PAGE, and the band corresponding to the 82kDa protein was excised from the gel. The collected protein was digested with lysyl endopeptidase at 37°C for 16h and then with trypsin at 37°C for 4h. The digested sample was resolved with reverse-phase HPLC, and two peptide fragments were selected for amino acid sequencing using the Edman degradation method (Toray Research Center, Inc., Kamakura, Japan).

### Purification and Detection of dsRNA

Viral dsRNA was extracted from 0.2g (dry weight) of fungal mycelia using a micro-spin column method (Okada et al., 2015). Briefly, virus particles were isolated as described above, and 0.2ml of viral suspension was mixed with 0.2ml of 2×STE buffer (20mm Tris–HCl pH 8.0, 2mm EDTA, 200mm NaCl) containing 1% SDS and 0.2ml of phenol:chloroform:isoamyl alcohol (25:24:1). The mixture was vortexed for 10min at room temperature and centrifuged at 15,000*g* for 5min, and the aqueous phase was collected. The purified dsRNA was subjected to agarose gel electrophoresis (1%) containing ethidium bromide (EtBr, 0.5μg/ml).

### Detection of 5' Cap Structures

We used RNA dot blot assays to look for m^7^G cap structures at the 5' ends of the AaV1 dsRNA segments (Supplementary Figure S2). The AaV1, ScV-L-A, and MyRV1 dsRNAs (1,000, 500, and 250ng/μl, respectively) were heat-denatured at 95°C for 5min and chilled on ice for 5min, and then, 1μl of each solution was spotted onto Zeta-Probe Membrane (Bio-Rad). After UV cross-linking twice with 120,000μJ/cm^2^ for 1min each time in a UV crosslinker (UVC500, Hoefer Inc., Holliston, MA), the membrane was agitated in 20ml blocking buffer containing 1×TBS-T buffer (0.02M Tris, 0.15M NaCl, 0.05% Tween-20, pH 7.4) and 5% skim milk powder at room temperature for 1h. Then, the membrane was gently rinsed in 10ml of 1×TBS-T buffer three times for 5min each. Subsequently, the membrane was probed in 10ml of primary antibody solution containing 1×TBS-T buffer, 1% skim milk powder, and 10μg of an anti-m^7^G-cap monoclonal antibody (mAb; Code No. RN016M, MBL^®^, Woburn, MA) at room temperature for 2h with gentle agitation. The membrane was rinsed three times as described above and then probed in 10ml of secondary antibody solution containing 1×TBS-T buffer, 1% skim milk powder, and 2μg of a goat anti-mouse IgG (Code No. 401215, Merck, Darmstadt, Germany) at room temperature for 1h with gentle agitation. After rinsing again, the antibody-bound spots were detected using the EzWestLumi plus and EZ-Capture MG system (ATTO, Japan).

We then used RNA ligase-mediated rapid amplification of the cDNA ends (RLM-RACE) with the GeneRacer^™^ Kit (Thermo Fisher Scientific, Waltham, MA) to confirm the presence of the 5' cap structures on each of the AaV1 dsRNA segments (Supplementary Figure S3). First, 500ng of AaV1 dsRNA was resuspended in 10μl distilled water; then, DMSO (90% v/v) was added and the RNAs were denatured at 65°C for 15min. Next, the denatured dsRNAs were recovered by ethanol precipitation and sequentially treated with calf intestinal phosphatase and tobacco acid pyrophosphatase, following the manufacturer’s protocol. The GeneRacer™ oligo RNA (Supplementary Table S1) was then ligated to the 5' ends of the treated dsRNAs using T4 RNA ligase. Oligo (dT)_36_ primers were used to create first-strand cDNA from the oligo RNA-ligated dsRNAs, and then, GoTaq^®^ Green Master Mix (Promega) was used in PCR with the GeneRacer™ 5' primer (Supplementary Table S1) and specific 3' reverse primers (Supplementary Table S1) to amplify the target fragments.

### Northern Hybridization Analysis

The AaV1 dsRNAs were separated by electrophoresis in 1% agarose gels, denatured in 10% (v/v) formaldehyde at 60°C for 1h, chilled in 20×SSC buffer for at least 15min, and then blotted onto nylon membranes (Zeta-Probe, Bio-Rad) using the capillary method. After cross-linking in a UV crosslinker (UVC500., Hoefer Inc), the membranes were probed with a digoxygenin (DIG) labeled DNA probe. The probe (499nt) was synthesized as a PCR product amplified from full-length AaV1 dsRNA2 using dsRNA2-specific primers (Supplementary Table S1). Northern hybridization was conducted using the DIG DNA Labeling and Detection Kit (Roche) following the manufacturer’s protocols.

### RT-PCR, Cloning, and Sequencing

AaV1 dsRNA was heat-denatured at 98°C for 5min and immediately chilled on ice for at least 5min. The SuperScript III First-strand synthesis system (Invitrogen) was used for first-strand cDNA synthesis, and then, PCR was performed using the KOD One™ PCR Master Mix (TOYOBO). The PCR conditions were as follows: 95°C for 3min followed by 35cycles of 95°C for 45s, 55°C for 30s, and 72°C for 45s. The PCR products were then analyzed by electrophoresis in 1% agarose gels containing EtBr (0.5μg/ml). The primer pairs used are listed in Supplementary Table S1.

After electrophoresis, the predicted PCR bands were extracted from the agarose gels and purified using the GENECLEAN II Kit (MP Biomedical). EX-*Taq* was used for A-tailing, and the PCR products were then cloned into the pCR™ 4-TOPO™ TA-cloning Vector (Invitrogen). The cloned PCR products were sequenced using the BigDye Terminator v3.1cycle sequencing kit (Applied Biosystems) and the Applied Biosystems 3130xl Genetic Analyzer (Applied Biosystems) according to the manufacturer’s protocols. The sequences were analyzed with MegAlign software (Lasergene7, DNA-STAR^®^, WI, United States).

## Results

### Curing *A. alternata* Isolate EGS 35–193 of AaV1

We hypothesized that AaV1 would propagate and be distributed heterogeneously in the EGS 35–193 hyphae (Supplementary Figure S1B). Therefore, AaV1-infected hyphae were cut into small fragments, spread out on YGA, and then screened for reduced virus titer or virus absence by dsRNA extraction followed by agarose gel electrophoresis and primer-specific RT-PCR.

Three isolates with reduced AaV1 titers were detected among fifteen isolates assessed (nos. 4, 7, and 10, Supplementary Figure S4A). One of them (no. 10) was selected, and the fragmentation process was repeated. Finally, we succeeded in obtaining two isolates cured of AaV1 among ten isolates assessed (nos. 10–9 and 10–10, Supplementary Figure S4B), as confirmed by RT-PCR using specific primer sets for each of the four dsRNA segments (Supplementary Figure S4C). We confirmed the stability of virus-free isolates by primer-specific RT-PCR after subculturing several times (data not shown).

### Presence of 5' Cap Structures on AaV1 dsRNAs

**Figure 1:**
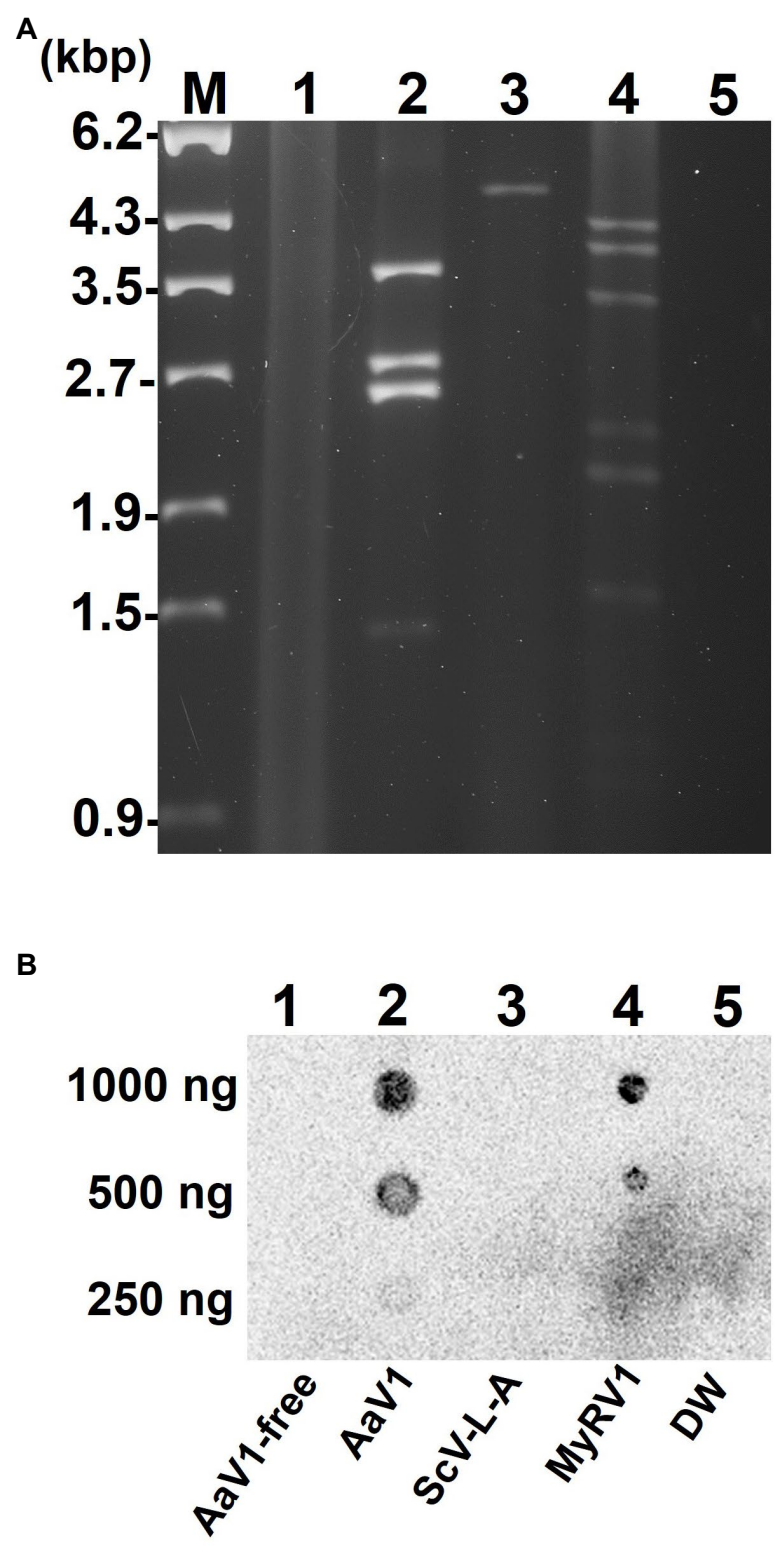
Detection of the 5' cap structures in the AaV1 dsRNA genome by RNA dot blot with the anti-m^7^G-Cap mAb. **(A)** Viral dsRNAs from purified virus particles. The dsRNAs were isolated from virus particles and then electrophoresed in 1% agarose gel with EtBr (0.5μg/ml) at 18V for 20h. Lane designation: M, 250ng of λ-EcoT14I-digested DNA marker; 1, AaV1-free; 2, AaV1 dsRNAs; 3, ScV-L-A dsRNA; 4, MyRV1 dsRNAs; 5, DW (distilled water, no template control). **(B)** RNA dot blot assay with the anti-m^7^G-Cap mAb. The dsRNA solutions (1,000, 500, and 250ng/μl) were dropped on the nylon membrane then probed with anti-m^7^G-Cap mAb. The AaV1 dsRNAs and the MyRV1 dsRNAs (positive control) showed positive signals, while the AaV1-free sample, the ScV-L-A dsRNA (negative control) and DW showed no signal.

Viral dsRNA molecules were extracted from purified AaV1 (Supplementary Figure S5), ScV-L-A (Supplementary Figure S6), and MyRV1 (Supplementary Figure S7) virions (Figure 1A). Following quantification, an RNA dot blot assay with anti-m^7^G-Cap mAb was performed for detecting the presence of 5' cap structures. The dsRNA dots of AaV1 and MyRV1 (positive control) showed signals, while the dots from an AaV1-free isolate, ScV-L-A, and DW (negative and no template controls) showed no signal (Figure 1B).

**Figure 2:**
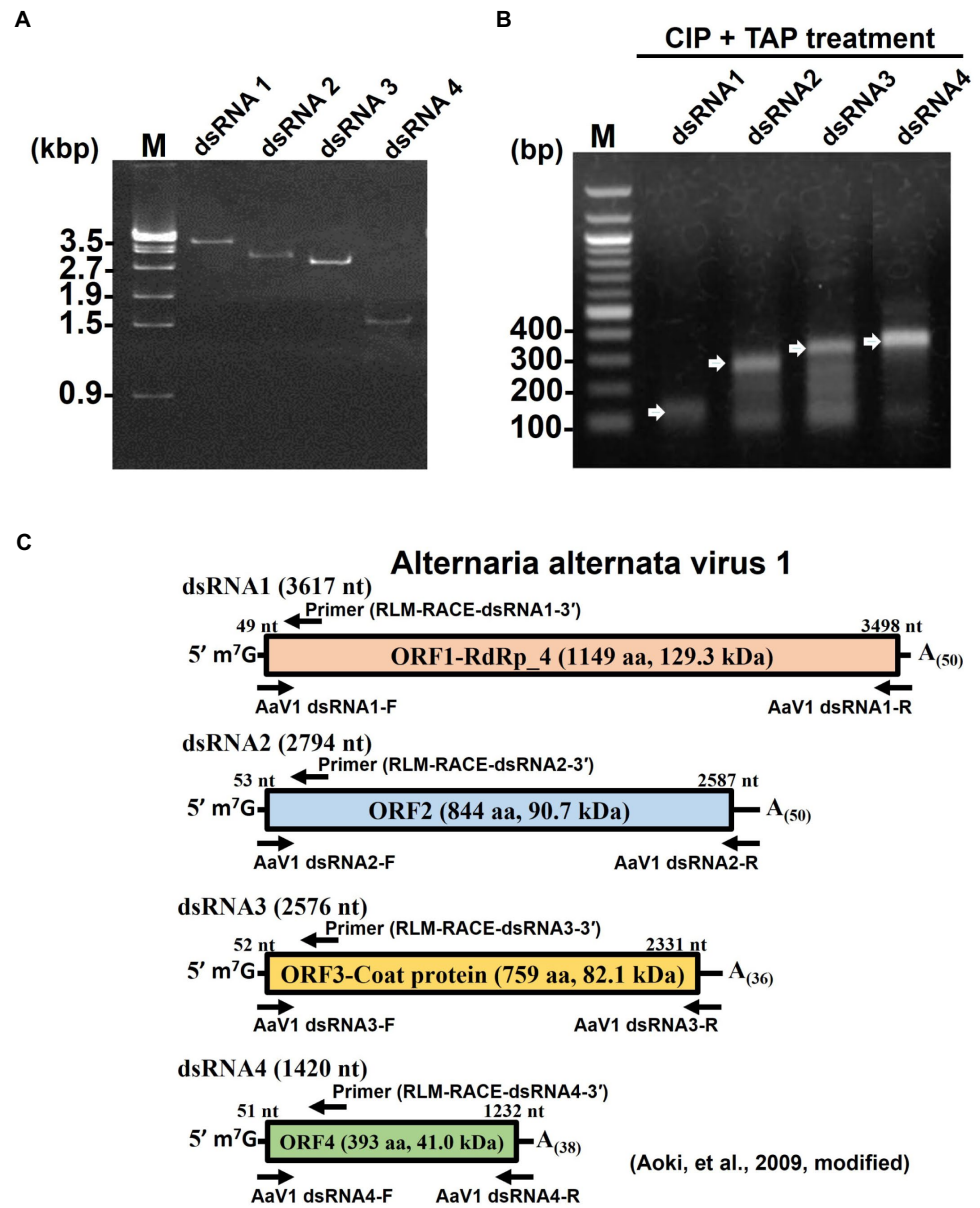
Detection of 5' cap structures on each AaV1 dsRNA segment by RLM-RACE. **(A)** Electrophoresis of the separately excised AaV1 dsRNA segments in a 1% agarose gel with EtBr (0.5μg/ml) at 50V for 1h (Mupid-2plus, Takara Bio, Japan). **(B)** Results of the RLM-RACE analysis, confirming the presence of 5' cap structures on each AaV1 dsRNA segment. The separately excised dsRNAs were subjected to the RLM-RACE procedure (Supplementary Figure S3) and then electrophoresed in a 1% agarose gel with EtBr (0.5μg/ml) at 100V for 0.5h. Lane M, 100bp DNA ladder. The arrows indicate the amplified target bands. **(C)** Diagrams of the AaV1 dsRNA1, 2, 3, and 4 segments showing the primer pairs used for RLM-RACE and for amplification of each full-length dsRNA segment.

To further investigate the 5' end structure of each AaV1 dsRNA, the four segments (Figure 1A, lane 2) were extracted from the agarose gel (Figure 2A) and used as templates in RLM-RACE experiments, which would yield amplicons only when dsRNA was capped (Supplementary Figure S3). For each segment, the results revealed amplicons of the predicted size based on the designed primers, as shown in Figure 2B: 108bp, 242bp, 279bp, and 307bp for dsRNA1, dsRNA2, dsRNA3, and dsRNA4, respectively (Supplementary Table S1; Figure 2C).

### Analysis of the AaV1 Major Structural Protein

**Figure 3:**
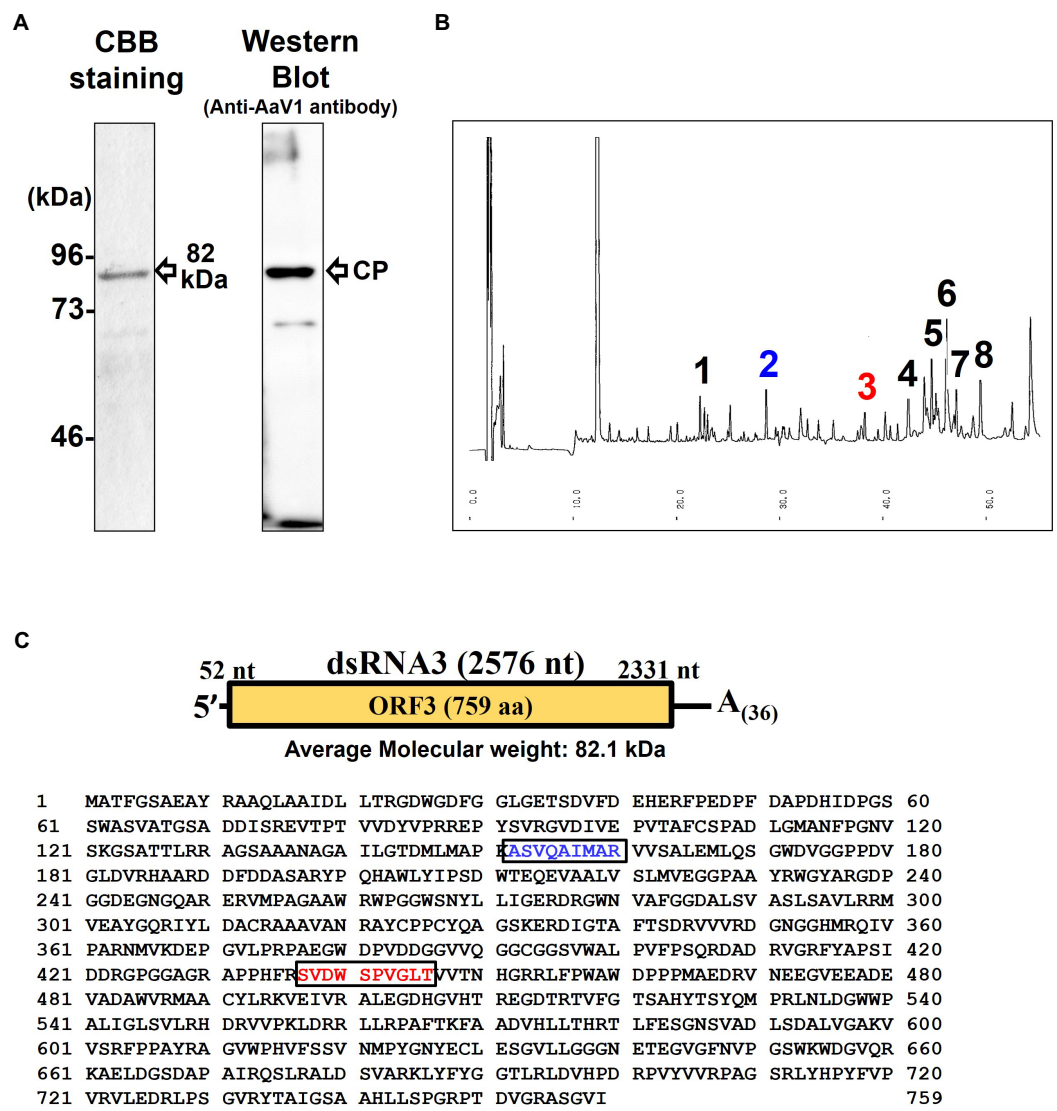
Characterization of the AaV1 major coat protein. **(A)** SDS-PAGE and western blot of the purified AaV1 particles. Purified viral proteins from strain EGS 35–193-1d were stained with CBB (left) or immunoblotted with antiserum against the AaV1 virus particles (right). The arrows indicate the viral structural protein. **(B)** Reverse-phase HPLC of the 82kDa major protein after digestion with Lysyl endopeptidase and trypsin. Peaks 2 and 3 were subjected to Edman degradation. **(C)** The deduced amino acid sequence of ORF3 (759 aa) written in one-letter code. The peptide sequences of peak 2 (blue) and peak 3 (red) were the same as the two regions in the predicted ORF3 peptide sequence.

The purified AaV1 particle proteins were used as antigens to immunize rabbits, and an IgG against AaV1 was purified from the anti-AaV1 antiserum. Western blot analysis of the purified AaV1 virions showed that the purified anti-AaV1 IgG specifically detected an 82kDa protein band and also visible following SDS-PAGE with CBB staining (Figure 3A). In our former report on AaV1 (Aoki et al., 2009), we estimated the molecular weight of the AaV1 major protein at 97kDa; however, our further analysis clarified that the major protein band was approximately 82kDa.

Edman degradation was used to investigate the primary structure of the purified 82kDa protein and to clarify which dsRNA segment encoded the AaV1 structural protein. Since no phenylthiohydantoin-amino acid derivatives were observed after five cycles of reaction, we realized that the N-terminus of the 82kDa protein was blocked (data not shown). We then used in-gel digestions to treat the major protein band with lysyl endopeptidase and trypsin. This resulted in two clear peaks (peaks 2 and 3) in reverse-phase HPLC (Figure 3B). Both peak 2 (ASVQAIMAR, blue-colored) and peak 3 (SVDWSPVGLT, red-colored) corresponded to the internal sequences of the predicted protein encoded by dsRNA3 (Figure 3C).

### Morphological Effects of Variations in AaV1 dsRNAs in EGS 35–193 Isolates

**Figure 4:**
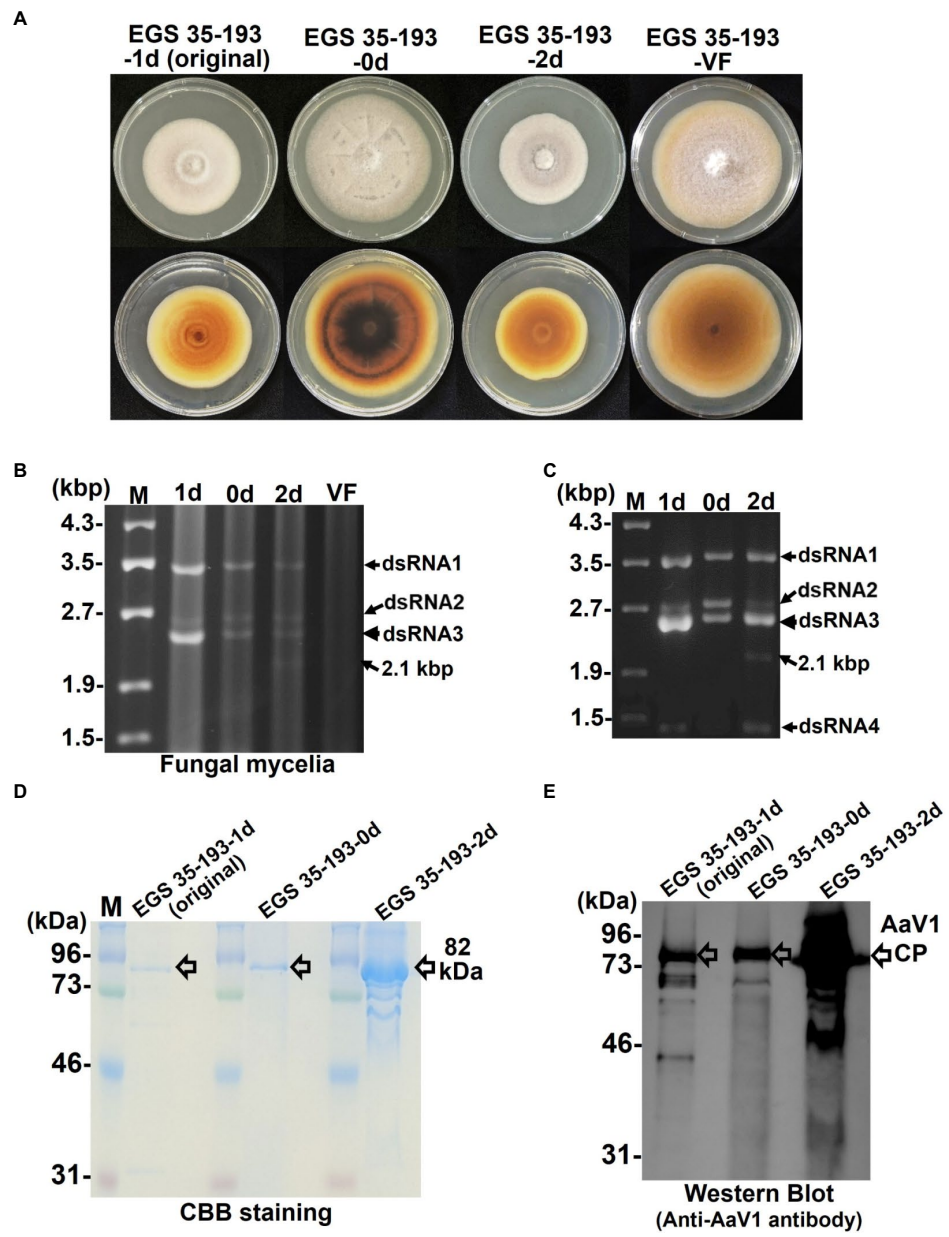
Phenotypic changes in EGS 35–193 mycelia caused by deletions of AaV1 dsRNA2. **(A)** Four types of colony morphologies were exhibited by the EGS 35–193 derivatives EGS 35–193-1d (the original strain), EGS 35–193-0d, EGS 35–193-2d, and EGS 35-195-VF (AaV1-free), grown on YGA plates for 7days at 25°C. (B, C) Agarose gel electrophoresis of dsRNAs purified from mycelia (20mg) of the four EGS 35–193 derivatives, purified by the spin column method **(B)**, and dsRNAs extracted from purified virus particles of the three EGS 35–193 AaV1-infected isolates **(C)**. The dsRNAs were separated in 1.0% agarose gels with EtBr (0.5μg/ml) at 18V for 20h. Lane designation: M, 250ng of λ-EcoT14I-digested DNA marker; 1d, EGS 35–193-1d; 0d, EGS 35–193-0d; 2d, EGS 35–193-2d; VF, EGS 35-193-VF. **(D)** SDS-PAGE of purified virus particles from EGS 35–193-1d, −0d, and-2d. The purified viral proteins were separated in an 8% polyacrylamide gel at 120V for 2h and then stained with CBB. Lane M, prestained protein marker. **(E)** Western blot analysis of purified virus particles from EGS 35–193-1d, −0d, and-2d, with antiserum raised against the AaV1 virus particles from EGS 35–193-1d.

During subculturing of the original AaV1-infected *A. alternata* EGS 35–193-1d on YGA plates, we noted sectors with distinct mycelial morphology: one with a flatter mycelium and intense pigmentation (named EGS 35–193-0d) and the other with slow growth (named EGS 35–193-2d; Figure 4A). Compared with the virus-free isolate of EGS 35–193 (named EGS 35-193-VF), the three AaV1-infected isolates showed impaired and abnormal growth phenotypes. EGS 35–193-0d showed the highest growth rate among the three AaV1-infected isolates, followed by EGS 35–193-1d, and lastly by EGS 35–193-2d (Supplementary Figure S8). High levels of pigmentation could only be observed in EGS 35–193-0d, while the other AaV1-infected isolates and the virus-free isolate showed modest pigmentation on YGA plates (Figure 4A). These different phenotypes were also seen when the isolates were grown on other media (Supplementary Figure S9).

To confirm the presence of AaV1 in the three EGS 35–193 variants, we purified dsRNAs from the fungal mycelia (Figure 4B) and from virions isolated from the mycelia of the three variants (Figure 4C; Supplementary Figures S5, S10). The purified virions were analyzed by SDS-PAGE and with a western blot probed with the anti-AaV1 antibody (Figures 4D,E). Although all three AaV1-infected isolates harbored the four dsRNA elements, the ratio of dsRNA2 (2,794bp) to dsRNA3 (2,576bp) fluctuated in preparations from both the fungal mycelia and the virions (Figures 4B,C). In preparations from EGS 35–193-0d, the dsRNA2 and dsRNA3 bands had similar intensities, while the dsRNA3 bands were much more intense than the dsRNA2 bands in preparations from both EGS 35–193-1d and EGS 35–193-2d (Figures 4B,C). In addition, a 2.1 kbp dsRNA was discovered in EGS 35–193-2d (Figures 4B,C, lane 2d, black arrows). We investigated the origin of the 2.1 kbp dsRNA in the following experiments.

### Detection of Defective dsRNA2 Segments in AaV1-Infected *A. alternata*

**Figure 5:**
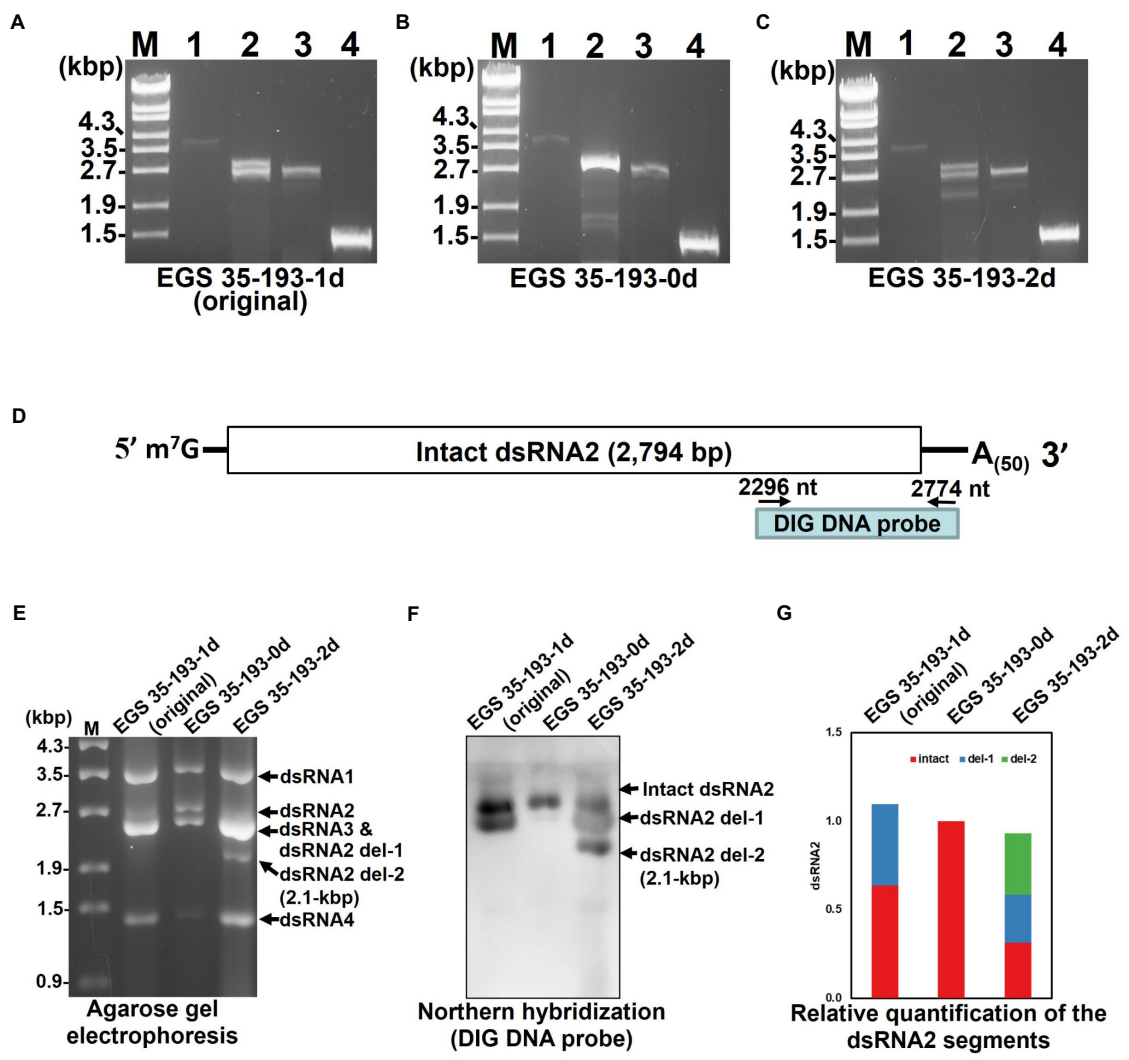
Analysis of the AaV1 dsRNA2-associated segments in virons purified from the three AaV1-infected isolates. (A-C) RT-PCR detection of the full-length dsRNA1–4 segments in EGS 35–193-1d **(A)**, EGS 35–193-0d **(B)**, and EGS 35–193-2d **(C)**. RT-PCR was performed with the four primer pairs (Figure 2C; Supplementary Table S1) designed to amplify the full-length dsRNA1–4 segments. The RT-PCR products were separated in 1.0% agarose gels with EtBr (0.5μg/ml) at 50V for 1h. Lane designation: M, 250ng of λ-EcoT14I-digested DNA marker; 1, dsRNA1; 2, dsRNA2; 3, dsRNA3; 4, dsRNA4. **(D)** Position of the DIG DNA probe used to detect the 3' region of AaV1 dsRNA2 in the northern hybridization experiment. The DIG DNA probe was synthesized using the probe synthesis primer pair (Supplementary Table S1). **(E)** Agarose gel electrophoresis of dsRNA genomes extracted from the purified virus particles of the three AaV1-infected isolates. These AaV1 dsRNAs were separated in a 1.0% agarose gel with EtBr (0.5μg/ml) at 18V for 20h. Lane M, 250ng of λ-EcoT14I-digested DNA marker. **(F)** Northern hybridization to detect the AaV1 dsRNA2-associated segments. After agarose gel electrophoresis, the dsRNA genomes were denatured and blotted onto a nylon membrane, and probed with the DIG DNA probe. **(G)** Relative quantification of the three dsRNA2 segments, intact, del-1 and del-2, following northern hybridization (F). The total signal in each lane was normalized using the amount of dsRNA 1, following agarose gel electrophoresis (E). Quantification of individual bands was performed using Fiji/ImageJ.

To explain the varying amounts of dsRNA2 and dsRNA3 in the three EGS 35–193 isolates and to identify the origin of the 2.1 kbp dsRNA segment in EGS 35–193-2d (Figures 4B,C), we conducted RT-PCR with specific primers designed to amplify the full length of each AaV1 segment excluding the poly(A) tail (Figure 2C; Supplementary Table S1). We used dsRNAs from AaV1 virions isolated from each EGS 35–193 isolate as templates. In the sample from EGS 35–193-0d, we obtained full-sized PCR bands derived from dsRNA1 (3.6 kbp), dsRNA2 (2.8 kbp), dsRNA3 (2.6 kbp), and dsRNA4 (1.4 kbp; Figure 5B, lanes 1–4). However, in the samples from EGS 35–193-1d and EGS 35–193-2d, we obtained multiple PCR bands, ranging from 2.1–2.8 kbp, with the dsRNA2-specific primer pair (Figures 5A,C, lane 2). These results suggested that the fluctuating ratios of AaV1 dsRNA2 to dsRNA3 and the 2.1-kbp dsRNA segment may be attributed to these dsRNA2 variants, which potentially carried internal deletions. To confirm this hypothesis, northern hybridization was performed using dsRNAs from each AaV1-infected isolate with a DIG-labeled dsRNA2-specific probe (Figure 5D; Supplementary Table S1). Only one signal corresponding to the intact, 2,794bp dsRNA2 was detected in the EGS 35–193-0d isolate (Figures 5E,F). In the EGS35-193-1d isolate, we detected two signals corresponding to the intact dsRNA2 (2,794bp) and dsRNA2 del-1, similar in size to the dsRNA3 segment (2,576bp; Figures 5E,F). In the EGS 35–193-2d isolate, we detected three signals corresponding to the intact dsRNA, dsRNA2 del-1, and dsRNA2 del-2, which was similar in size to the additional 2.1 kbp segment detected by agarose gel electrophoresis, were observed (Figures 5E,F).

### Characterization of the Deletions in AaV1 dsRNA2 del-1 and dsRNA2 del-2

**Figure 6:**
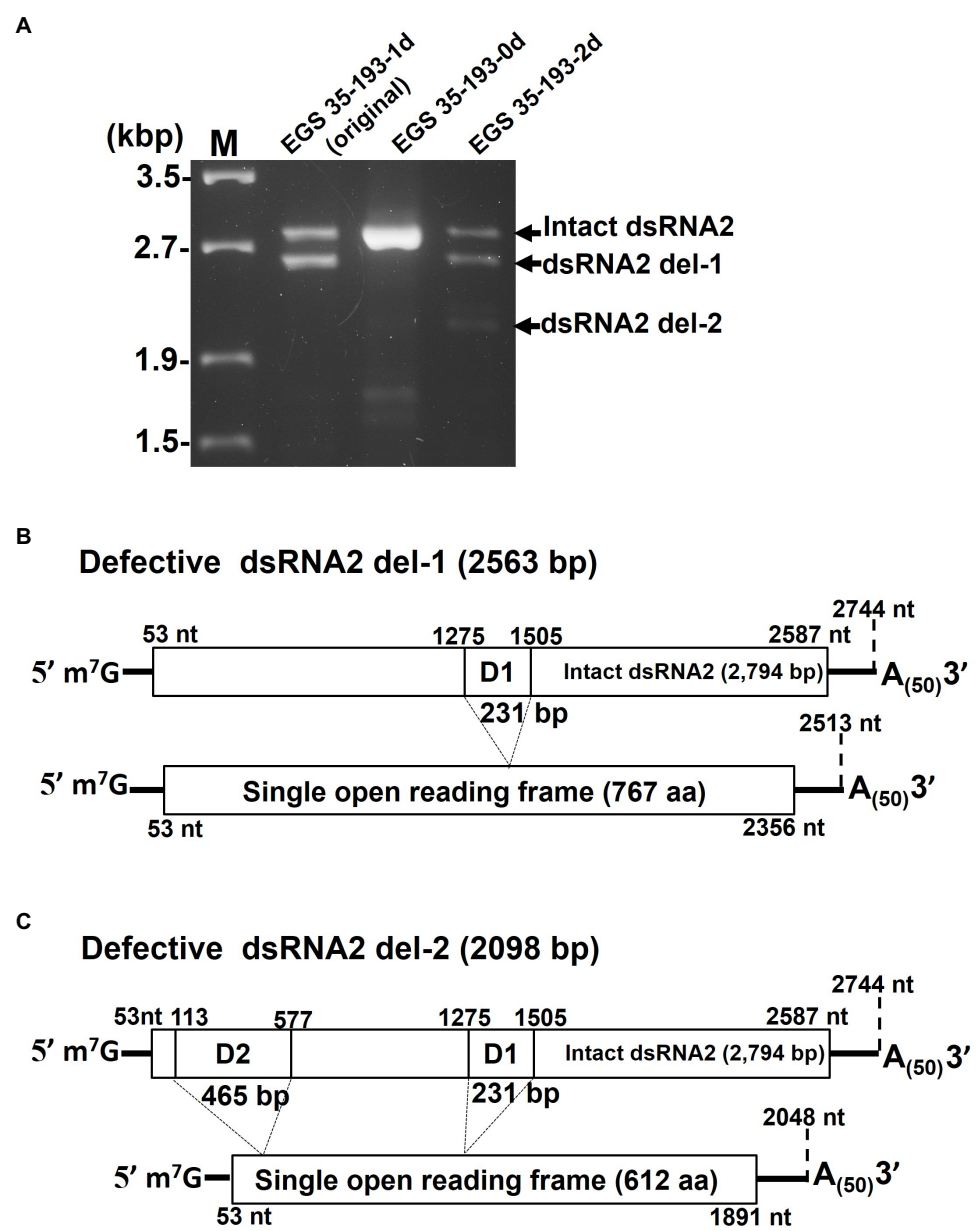
Determination of the deleted regions in the dsRNA2 segments of AaV1. **(A)** Agarose gel electrophoresis of RT-PCR products amplified with the primer pair AaV1 dsRNA2-F and AaV1 dsRNA2-R, which is designed to amplify full-length AaV1 dsRNA2. The 1.0% agarose gel with EtBr (0.5μg/ml) was run at 18V for 20h. Lane M, 250ng of λ-EcoT14I-digested DNA marker. (B, C) Schematic diagrams showing the genome deletions of the AaV1 dsRNA2 derivatives. **(B)** Defective dsRNA2 del-1 has an in-frame deletion site (D1, 231bp) located at nt 1,275–1,505 of the intact AaV1 dsRNA2. **(C)** Defective dsRNA2 del-2 has two in-frame deletion sites, D1 and D2 (465bp), located at nt 113–577 of the intact AaV1 dsRNA2.

In order to identify the deleted regions in dsRNA2 del-1 and dsRNA2 del-2, we sequenced the PCR products shown in Figure 6A. The two amplified dsRNA2 products from EGS 35–193-1d, the intact dsRNA2 and the dsRNA2 del-1, were separated by extended electrophoresis (1% agarose, 18V, 20h; Figure 6A), extracted from the gel, cloned, and sequenced. We found an in-frame deletion site (D1) near the middle of the dsRNA2 segment: The region from nt 1,275 to nt 1,505 in the intact dsRNA2 was deleted in dsRNA2 del-1. The exact size of the dsRNA2 del-1 segment was 2,513bp excluding the poly(A)_50_, and the length of D1 was 231bp (Figure 6B and Supplementary Figure S11A).

A similar analysis was performed for EGS 35–195-2d (Figure 6A). The dsRNA2 del-1 segment from EGS 35–195-2d had only one deletion site, D1, which was identical to that in dsRNA2 del-1 from EGS 35–193-1d (Figure 6C; Supplementary Figure S11B). The dsRNA2 del-2 segment had two deletions: D1, which again was identical to the deletion in EGS 35–193-1d, and D2, a deletion in the 5' region of the segment spanning from nt 113 to nt 577 in the intact dsRNA2 (Figure 6C; Supplementary Figure S11B). The D1 and D2 deletions shortened the length of dsRNA2 del-2 from 2,794bp to 2098bp. Both the defective dsRNA2 del-1 and dsRNA2 del-2 segments had open reading frames encoding putative proteins of 767 aa and 612 aa, respectively (Figures 6B,C). The full-sized dsRNA2 segment had an open reading frame encoding a putative protein of 844 aa (Figure 6A).

## Discussion

As we reported previously, AaV1 was the first dsRNA virus to be identified with a poly(A) tail at the 3' end of each segment of its genome (Aoki et al., 2009). The 5' m^7^G-cap structures were first found on the viral dsRNAs of vaccinia virus (Wei and Moss, 1975) and cytoplasmic polyhedrosis virus, belonging to the family *Reoviridae* (Furuichi and Miura, 1975). In this study, we used RNA dot blot assays and RLM-RACE to demonstrate that each of the AaV1 dsRNA segments has a capping structure, 7-methylguanosine (m^7^G), at its 5' end. To the best of our knowledge, AaV1 is the first dsRNA virus to be discovered with both the 3' poly(A) tail and the 5' cap structure on each genomic segment.

Many RNA viruses such as potexviruses, benyviruses, cucucmoviruses, tobamoviruses and reoviruses employ the cap-dependent translation. Other RNA viruses employ the cap-independent translation; it is well known potyviruses use a viral protein (VPg) that is covalently linked to the 5' end of the RNA (Zhang et al., 2015), and carmoviruses use the 3'-untranslated region (Simon, 2015) as alternatives for assisting the formation of the translation initiation complex. The influenza virus and the yeast L-A virus have mechanisms for snatching cellular mRNA caps and using them to assist translation of the viral RNA (Plotch et al., 1979; Fujimura and Esteban, 2011). The cap at the 5' end is essential for translation initiation of cellular mRNAs and is associated with the eucaryotic initiation factors eIF4E, eIF4G, and eIF4A, which recruit the 40S ribosomal subunit (Lindqvist et al., 2008). In addition, the cap structure also serves as a defense against *SKI*/*XRN1* exoribonuclease, which explicitly degrades mRNAs with no cap (Masison et al., 1995). Thus, the cap structures of AaV1 are considered advantageous for effective viral propagation.

The 5' cap structure and the 3' poly(A) tail have the functions of protecting the genome, transporting the RNA, and enhancing translation (Hocine et al., 2010). Cap structures also increase the accuracy and efficiency of mRNA splicing, which generally occurs in the nucleus (Inoue et al., 1989). It seems more efficient for mycoviral RNA genomes to have a 5' cap and a 3' poly(A) tail for propagation in the cytoplasm of eukaryotic cells, since these modifications act synergistically to enhance translation (Gallie, 1991). However, few RNA viruses have a cap and a poly(A) tail, and the reason for this is unknown. Indeed, ribosomal RNA, which accounts for 60% of the total RNA in cells (Woolford Jr and Baserga, 2013), also does not have a cap and a poly(A) structure and is not translated. It may be favorable for the mycoviruses to propagate in the cytoplasm without these terminal structures as this might lead to high copy numbers.

In our previous study, the viral proteins were analyzed by 7% SDS-PAGE and the protein size was determined as 97kDa by comparison with the low molecular weight marker (GE Healthcare, United Kingdom; Aoki et al., 2009). However, in this study, we analyzed the viral proteins by 10% SDS-PAGE and found that the size of the major protein was 82kDa, using DynaMarker^®^ Protein MultiColor (Funakoshi Co., Ltd., Japan). This corresponds with the predicted size of the protein encoded by dsRNA3. Edman degradation demonstrated that the AaV1 dsRNA3 encodes the 82kDa protein, which is the major structural protein of the AaV1 virion. While carrying out the Edman analysis, we found that the N-terminus of the 82kDa protein was blocked (data not shown). It is possible that the AaV1 82kDa protein is N-terminally acetylated since this is one of the most common protein modifications in eukaryotes (Arnesen et al., 2009). *MAK3* N-acetyltransferase modification of Gag is necessary for virion assembly of the yeast L-A virus (Tercero and Wickner, 1992; Tercero et al., 1993). N-terminal acetylation may also be necessary for AaV1 virion formation.

During the subculturing of the AaV1-infected EGS 35–193 strain, we found fungal isolates carrying virions with defective dsRNA2 segments, dsRNA2 del-1 and dsRNA2 del-2, which occurred by in-frame deletion events (Figures 5, 6). Based on agarose gel electrophoresis of the AaV1 dsRNA genomes purified from virions (Figure 4C), the defective dsRNA2 segments appeared to have no significant effects on accumulation of the other dsRNA segments. To confirm the relative quantification of dsRNA2 segments in three isolates, we analyzed the results of agarose gel electrophoresis (Figure 5E) and northern hybridization (Figure 5F), by Fiji/ImageJ software (Schneider et al., 2012). The results showed that the relative quantification of dsRNA2 segments in three isolates has no significant difference (Figure 5G), and the detailed results will be obtained by real-time RT-PCR in the future. The defective dsRNA2 segments were responsible for altered phenotypes in the fungal host, including reduced hyphal growth rates and irregular pigmentation (Figure 4A). No deletions were found in dsRNA1, dsRNA3, or dsRNA4. We speculate that this is because the AaV1 open reading frame ORF1 (RdRp) and ORF3 (coat protein) are essential for viral replication or virion packaging. Similar selective deletion events were found in mycoreoviruses. The inducible genome deletions of MyRV1 dsRNA S4 and S10 are related to changes in vertical transmission efficiency and host colony morphology, but not to viral replication (Sun and Suzuki, 2008; Eusebio-Cope et al., 2010; Kanematsu et al., 2014). Several other findings of RNA genome deletions in the hypoviruses also demonstrated their encoded in-frame fusion proteins affecting host growth negatively (Hillman et al., 2000; Xie et al., 2011; You et al., 2019).

Interestingly, both the deletions in AaV1 dsRNA2 were in-frame (Figures 6B,C). These in-frame deletions might have protected the deleted dsRNA2 segments from nonsense-mediated mRNA decay, since AaV1 dsRNAs with 5' cap structures would recruit decapping enzymes, such as Dcp1p/Dcp2, and the major cytoplasmic 5'-3' exonuclease (Ski1/Xrn1), which target nonsense-containing mRNAs (Peltz et al., 1993; Maderazo et al., 2003; Celik et al., 2017). Similar to the full-length ORF2, the two defective ORF2 segments contained no conserved domains based on searches of the NCBI database (data not shown). These in-frame fusion ORF2 proteins negatively affect host growth, but the exact mechanisms by which they effect host growth remain unclear. In the future, we will investigate the connection between the defective genomes and host growth using a heterologous expression system in yeast.

In our previous phylogenetic analysis, AaV1 was shown to be related to *Chrysoviridae* and *Totiviridae* (Aoki et al., 2009). The increased number of available mycovirus sequences has now allowed us to construct a more precise phylogenetic tree (Supplementary Figure S12A; Supplementary Table S2). The new tree shows that the Alternaviridae family is more closely related to *Totiviridae* than to *Chrysoviridae*. Interestingly, AaV1 and the other six alternaviruses have an ADD motif instead of GDD in the conserved motif VI of RdRp (Supplementary Figure S12B; Kamer and Argos, 1984; Koonin, 1991; Aoki et al., 2009). The GDD motif sometimes shows flexible glycine residue requirements, such as IDD in infectious bursal disease virus or SDD in phage Φ6, even though these variants possessed the same function as the GDD motif (Shwed et al., 2002). Therefore, we expect that the ADD sequence of RdRp motif VI of alternaviruses is functionally active.

In the future, we would like to investigate what advantages are provided by the cap and poly(A) structures on the AaV1 dsRNA genomic segments. The *SKI* genes are involved in 5'-3' and 3'-5' mRNA degradation pathways (Toh-E et al., 1978; Widner and Wickner, 1993; Zhang et al., 2019). Therefore, we will utilize *SKI*-deficient mutants or *SKI*-overexpressing strains of *Saccharomyces cerevisiae*, since this might provide valuable insights into the roles of the cap and poly(A) structures in RNA degradation.

## Data Availability Statement

The original contributions presented in the study are included in the article/Supplementary Material, and further inquiries can be directed to the corresponding author.

## Author Contributions

CW, NA, and NT performed the experiments with academic and technical assistance from TA, TF, RO, and HC. CW, KK, IK, and HM analyzed the data and wrote the first draft of the manuscript. All authors critically reviewed the manuscript and approved the final submission.

## Funding

This work was supported by a Grant-in-Aid for Challenging Exploratory Research from the Japan Society for the Promotion of Science (19K05946 and 20KK0137) to HM.

## Conflict of Interest

The authors declare that the research was conducted in the absence of any commercial or financial relationships that could be construed as a potential conflict of interest.

## Publisher’s Note

All claims expressed in this article are solely those of the authors and do not necessarily represent those of their affiliated organizations, or those of the publisher, the editors and the reviewers. Any product that may be evaluated in this article, or claim that may be made by its manufacturer, is not guaranteed or endorsed by the publisher.
