# Supplementary material for: Unique Terminal Regions and Specific Deletions of the Segmented Double-Stranded RNA Genome of Alternaria Alternata Virus 1, in the Proposed Family Alternaviridae
Source: Front Microbiol. 2021 Oct 22;12:773062. doi: 10.3389/fmicb.2021.773062 (PMC8570381; doi:10.3389/fmicb.2021.773062)
Supplement: Supplementary file 1 [file Data_Sheet_1.PDF]

Supplementary Material

Supplemental Table S1. List of the primers used in this study.

Primer name	Sequence	Purpose
GeneRacer™ oligo	5′-rCrGrArCrUrGrGrArGrCrArCrGrArGrGrArCrArCrUrGrArC rArUrGrGrArCrUrGrArArGrGrArGrUrArGrArArA	RLM-RACE of 5′ cap detection
GeneRacer™ 5′ primer	5′-CGA CTG GAG CAC GAG GAC ACT GA	
RLM-RACE-dsRNA1-3′	5′-TTC CTG AAC CCC AGC AAT AGG	
RLM-RACE-dsRNA2-3′	5′-ATG GGG AAA CGG GAA CGC C	
RLM-RACE-dsRNA3-3′	5′-AAA TGT CAT CGG CCG AAC CC	
RLM-RACE-dsRNA4-3′	5′-TTG TCA ACA TGG ACG TGA TGG	
AaV1 dsRNA1-F	5′-GAG GGA TCC TGG CAA AAA GCT ATC GTA TCT CG	AaV1 full-length genomes detection and cloning
AaV1 dsRNA1-R	5′-TTC GGA TCC GGA TAT TCC GAC TAA ATC ACG	
AaV1 dsRNA2-F	5′-CCG AAT TCC ACA AAG CAA TCG TAT CGC CAG	
AaV1 dsRNA2-R	5′-CGC GAA TTC ATA TTT GTT CCA CTA CAC CAG ACC	
AaV1 dsRNA3-F	5′-TGG AGC TCC ATA AAG CTA TCG TAT CTC GAG	
AaV1 dsRNA3-R	5′-TAC CGA GCT CGT TCC ACG TTC GAG ACA CGC	
AaV1 dsRNA4-F	5′-CCG AAT TCC ATA AAG CAA TCG TAT CGC CAG	
AaV1 dsRNA4-R	5′-GCT TGA ATT CGT TGT TAT CCT CAC AGC ACC	
AaV1 dsRNA2-F	5′-CCG AAT TCC ACA AAG CAA TCG TAT CGC CAG	DIG DNA probe synthesis
AAM2RA-C2	5′-TCT CGC GCA TAT GCG TGC TC	
AaV1-RdRp-F	5′-CTT AAC CGC GAG CTC TCG GCA A	AaV1 detection of curing experiment
AaV1-RdRp-R	5′-AGG CTC CAC AAC AAG CCT TGT A	
dsRNA2-check-5′	5′-TAG CAA GCG TGG GGT ATC G	
dsRNA2-check-3′	5′-ATT ACG GTA TTG AGT TGG C	
AaV1 ORF3-F	5′-ATA GTC GAC ATG GCG ACG TTT GGA AGT G	
AaV1 ORF3-R	5′-GAG TCG ACT CAA ATG ACA CCA GAA GCT C	
AaV1 ORF4-F	5′-GGG AAT TCA TGT TTG ATT CCT TTT GTT CC	
AaV1 ORF4-R	5′-AAG AAT TCT TAT GCA GAC GTA CCT ACT CC	

Supplemental Table S2. List of the amino acid sequences of viral RdRps used in the phylogenetic analysis of this study.

Virus name	Abbreviation	Accession No.
Chrysoviridae		
Alphachrysovirus		
Amasya cherry disease associated chrysovirus	ACDACV	YP_001531163
Anthurium mosaic-associated virus	AMAV	YP_009667023
Aspergillus fumigatus chrysovirus	AfuCV	YP_009508104
Brassica campestris chrysovirus 1	BcCV1	YP_009667006
Colletotrichum gloeosporioides chrysovirus 1	CgCV1	YP_009667012
Chrysothrix chrysovirus 1	CcCV1	QGR26538
Cryphonectria nitschkei chrysovirus 1	CnCV1	YP_009507942
Fusarium oxysporum chrysovirus 1	FoCV1	YP_009665200
Helminthosporium victoriae virus 145S	HvV145S	YP_052858
Isaria javanica chrysovirus 1	IjCV1	YP_009337840
Macrophomina phaseolina chrysovirus 1	MpCV1	YP_009667008
Penicillium chrysogenum virus	PcV	YP_392482
Persea americana chrysovirus	PaCV	YP_009666328
Raphanus sativus chrysovirus 1	RsCV1	YP_009667003
Shuangao insect-associated chrysovirus	SCLV	ASA47445
Verticillium dahliae chrysovirus 1	VdCV1	YP_009507948
Zea mays chrysovirus 1	ZMCV1	YP_009551655
Betachrysovirus		
Alternaria alternata chrysovirus 1	AaCV1	YP_009553287
Aspergillus thermomutatus chrysovirus 1	AthCV1	AWC67507
Botryosphaeria dothidea chrysovirus 1	BdCV1	YP_009353026
Colletotrichum fructicola chrysovirus 1	CfCV1	YP_009551629
Coniothyrium diplodiella chrysovirus 1	CdCV1	QDB74971
Fusarium graminearum dsRNA mycovirus 2	FgV2	ADW08802
Fusarium oxysporum f. sp. dianthi mycovirus	FodV	YP_009158913
Magnaporthe oryzae chrysovirus 1-A	MoCV1-A	YP_003858286
Neofusicoccum parvum chrysovirus 1	NpCV1	QDB74975
Penicillium janczewskii chrysovirus 1	PjCV1	YP_009182332
Penicillium janczewskii chrysovirus 2	PjCV2	YP_009667018

Virus name	Abbreviation	Accession No.
Totiviridae		
Totivirus		
Saccharomyces cerevisiae virus L-A	ScV-L-A	NP_620495
Saccharomyces cerevisiae virus L-BC	ScV-L-BC	NP_042581
Ustilago maydis virus H1	UmVH1	NP_620728
Scheffersomyces segobiensis virus L	SSVL	YP_009507831
Xanthophyllomyces dendrorhous virus L1A	XdV-L1A	YP_007697651
Xanthophyllomyces dendrorhous virus L1b	XdV-L1B	YP_009507835
Tuber aestivum virus 1	TaV1	YP_009507833
Victovirus		
Helminthosporium victoriae virus 190S	Hv190SV	NP_619670
Sphaeropsis sapinea RNA virus 1	SsRV1	NP_047558
Sphaeropsis sapinea RNA virus 2	SsRV2	NP_047560
Coniothyrium minitans RNA virus	CmRV	YP_392467
Magnaporthe oryzae virus 1	MoV1	YP_122352
Magnaporthe oryzae virus 2	MoV2	YP_001649206
Alternaria arborescens victorivirus 1	AaVV1	YP_009553478
Gremmeniella abientina RNA virus L1	GaRV-L1	NP_624332
Aspergillus foetidus slow virus 1	AfV-S1	YP_009508249
Beauveria bassiana victorivirus 1	BbVV1	YP_009508251
Chalara elegans RNA Virus 1	CeRV1	YP_024728
Helicobasidium mompa totivirus 1-17	HmV1-17	NP_898833
Rosellinia necatrix victorivirus 1	RnVV1	YP_008130308
Tolypocladium cylindrosporum virus 1	TcV1	YP_004089630
Leishmaniavirus		
Leishmania RNA virus 1-1	LRV1	NP_041191
Leishmania RNA virus 2-1	LRV2	NP_043465
Giardiavirus		
Giardia lamblia virus	GLV	NP_620070
Trichomonasvirus		
Trichomonas vaginalis virus 1	TVV1	AAA62868
Trichomonas vaginalis virus 2	TVV2	NP_624323
Trichomonas vaginalis virus 3	TVV3	NP_659390
Trichomonas vaginalis virus 4	TVV4	YP_009507836

Virus name	Abbreviation	Accession No.
Alternaviridae		
Alternavirus		
Alternaria alternata virus 1	AaV1	YP_001976142
Aspergillus mycovirus 341	AsV341	ABX79997
Aspergillus foetidus mycovirus	AfV-F	YP_007353985
Fusarium poae alternavirus 1	FpAV1	YP_009272952
Fusarium graminearum alternavirus 1	FgAV1	YP_009667012
Fusarium incarnatum alternavirus 1	FiAV1	AYJ09265
Aspergillus heteromorphus alternavirus	AheAV1	AZT88575

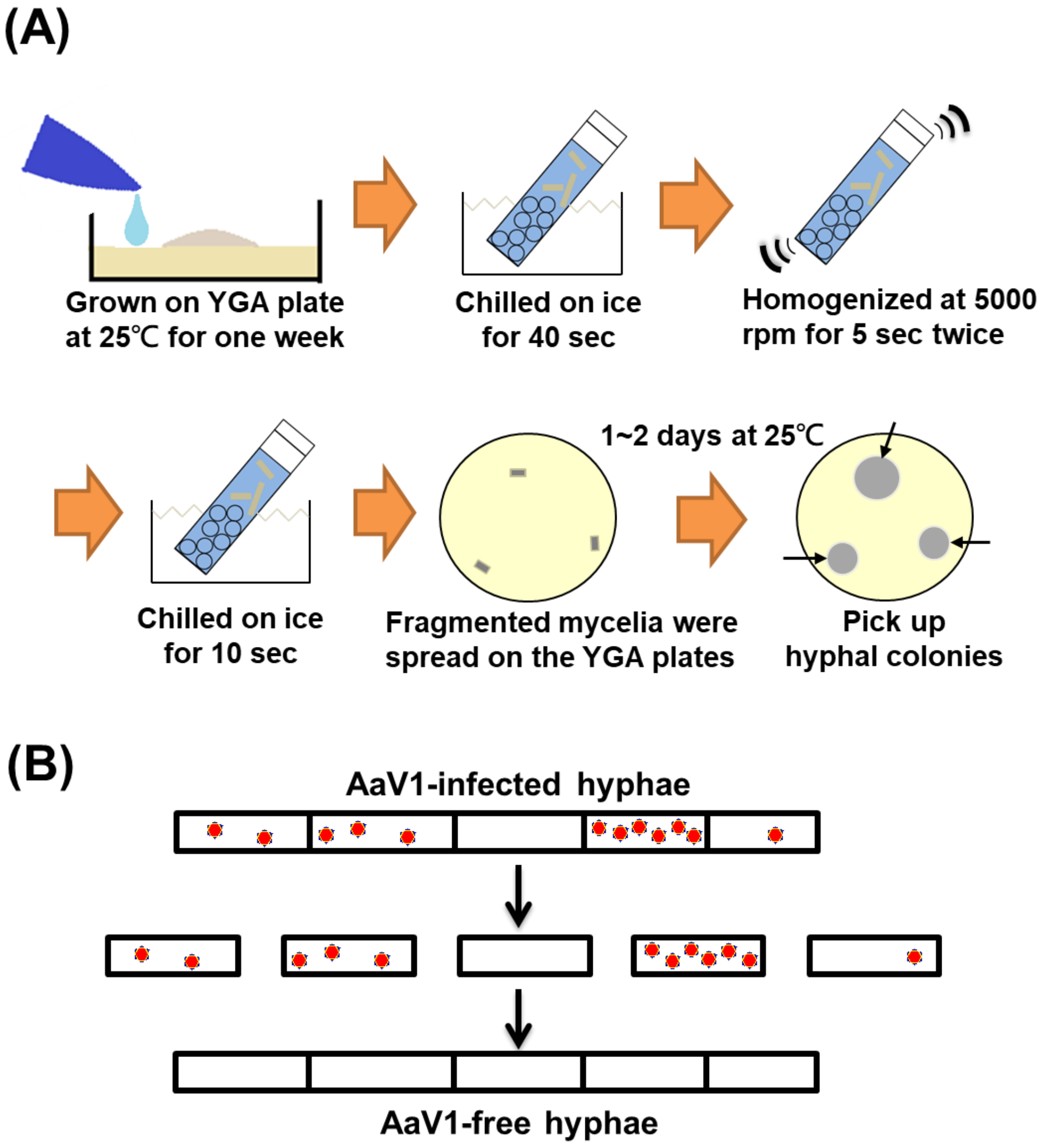

Supplemental Figure S1. Curing of AaV1-infected *A. alternata* isolate **(A)** Flowchart of procedures to isolate AaV1-free isolates, and **(B)** Paradigm of AaV1-distribution in hyphae of the strain EGS 35-193.

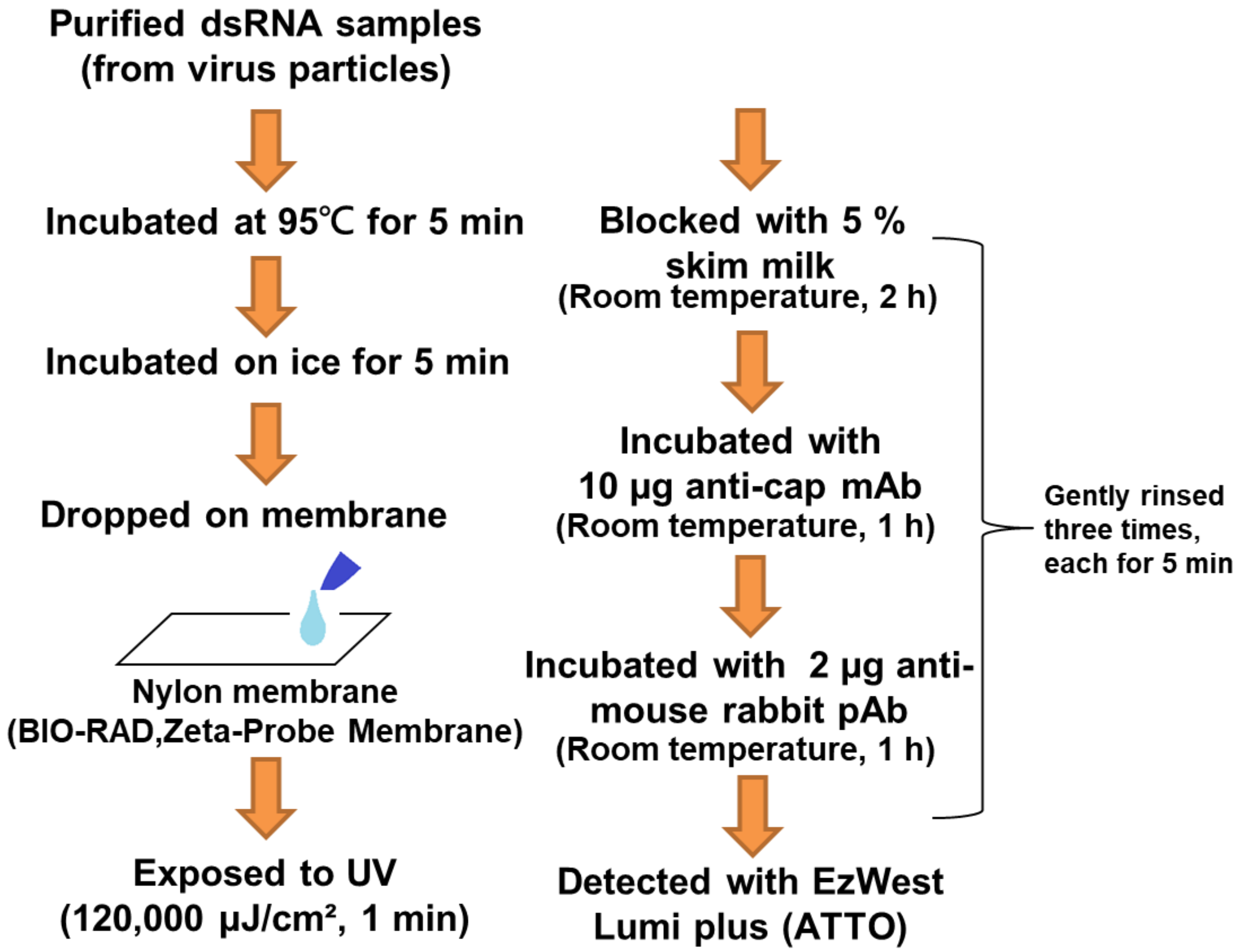

Supplemental Figure S2. Flowchart of RNA dot blot for detecting m^7G-cap structures in AaV1 dsRNA genomes.

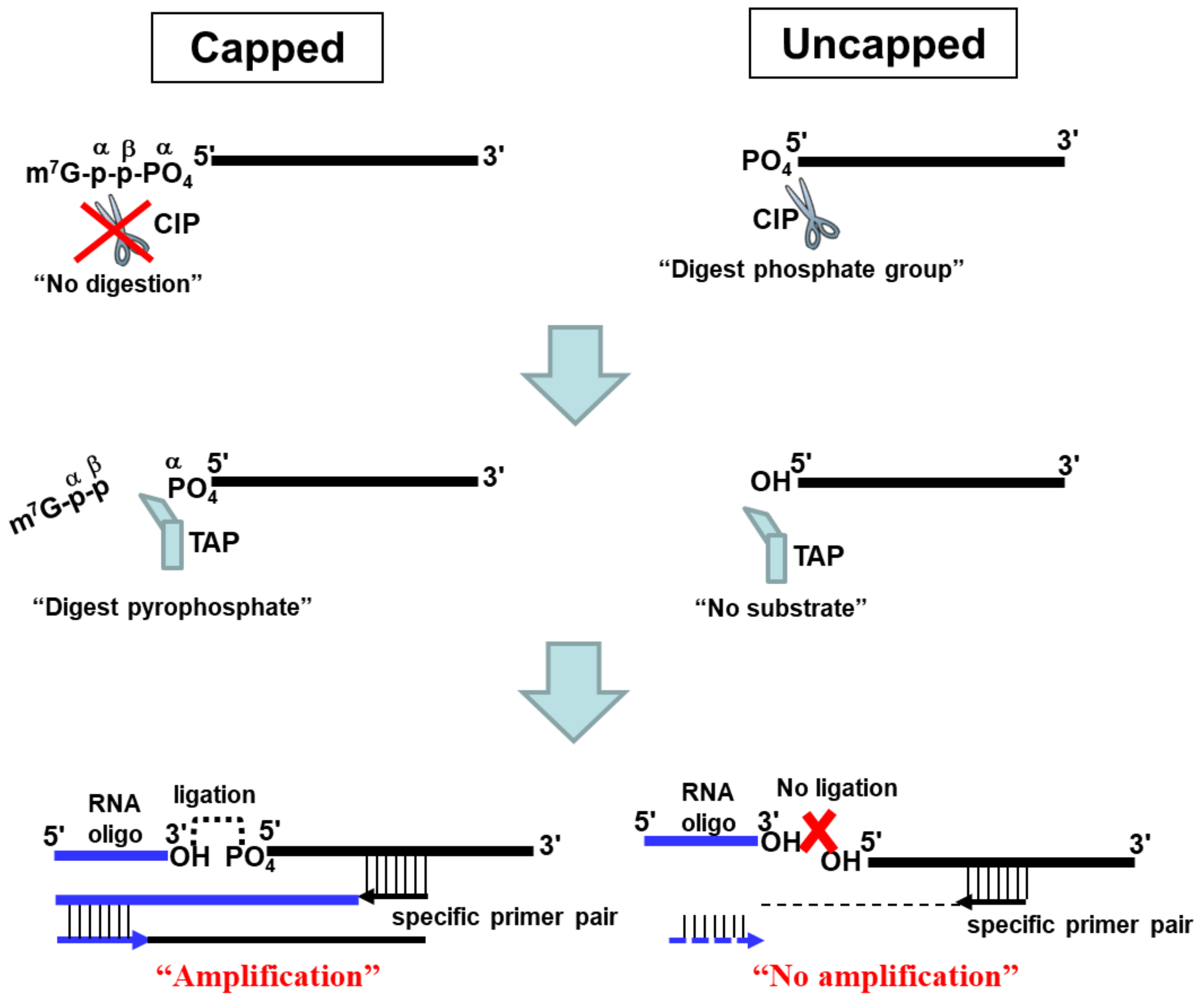

Supplemental Figure S3. Flowchart of RLM-RACE for detecting m^7G-cap structures in AaV1 dsRNA genomes.

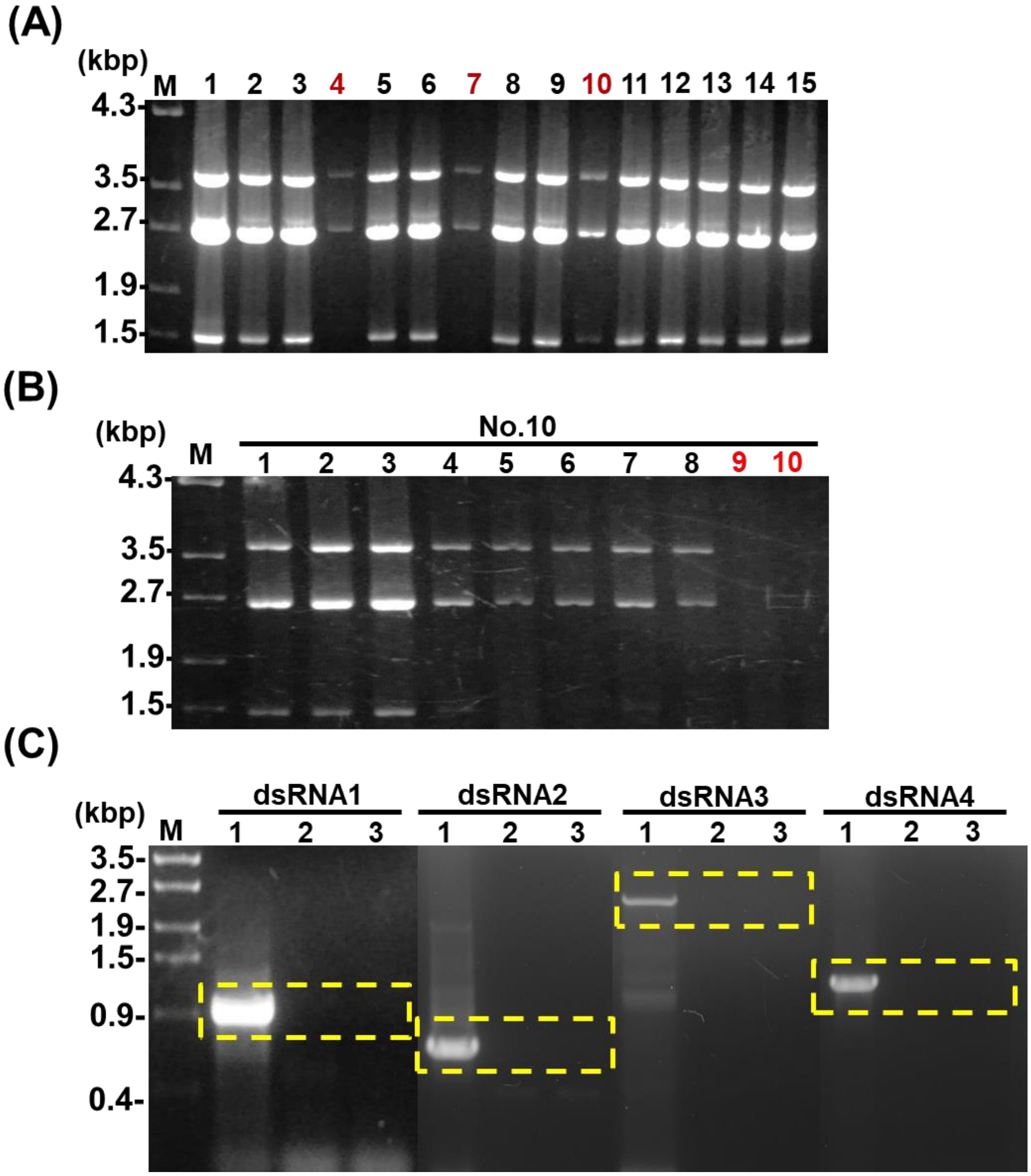

Supplemental Figure S4. Selection of AaV1-free isolates. **(A, B)** Step-wise selection of Aav1-free isolates after curing procedures. **(C)** The AaV1 specific RT-PCR bands were amplified from the AaV1 positive control (dsRNA1–937 bp; dsRNA2–733 bp; dsRNA3–2297 bp; dsRNA4–1198 bp), while no RT-PCR band was amplified from the isolates No.10-9 and No.10-10. These two isolates were used as AaV1-free isolates of EGS 35-193 in this study.

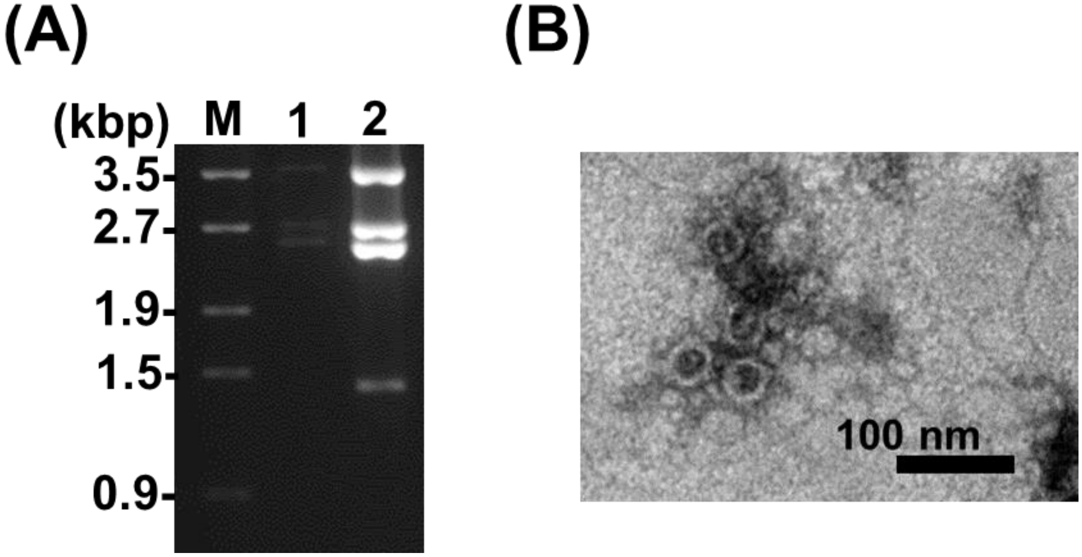

Supplemental Figure S5. Purified dsRNA genomes from virus particles of AaV1 (EGS 35-193-0d). **(A)** The dsRNAs were extracted from the fractions of 45% sucrose cushion in purification procedure. Lane designation: M, 250 ng of λ-EcoT14I-digested DNA marker; 1, supernatant fraction; 2, resuspended pellet fraction. **(B)** Purified virus particles of EGS 35-193 isolate. The purified virus particles from resuspended pellet fraction of 45% sucrose cushion were stained with 2% uranyl acetate and observed by TEM.

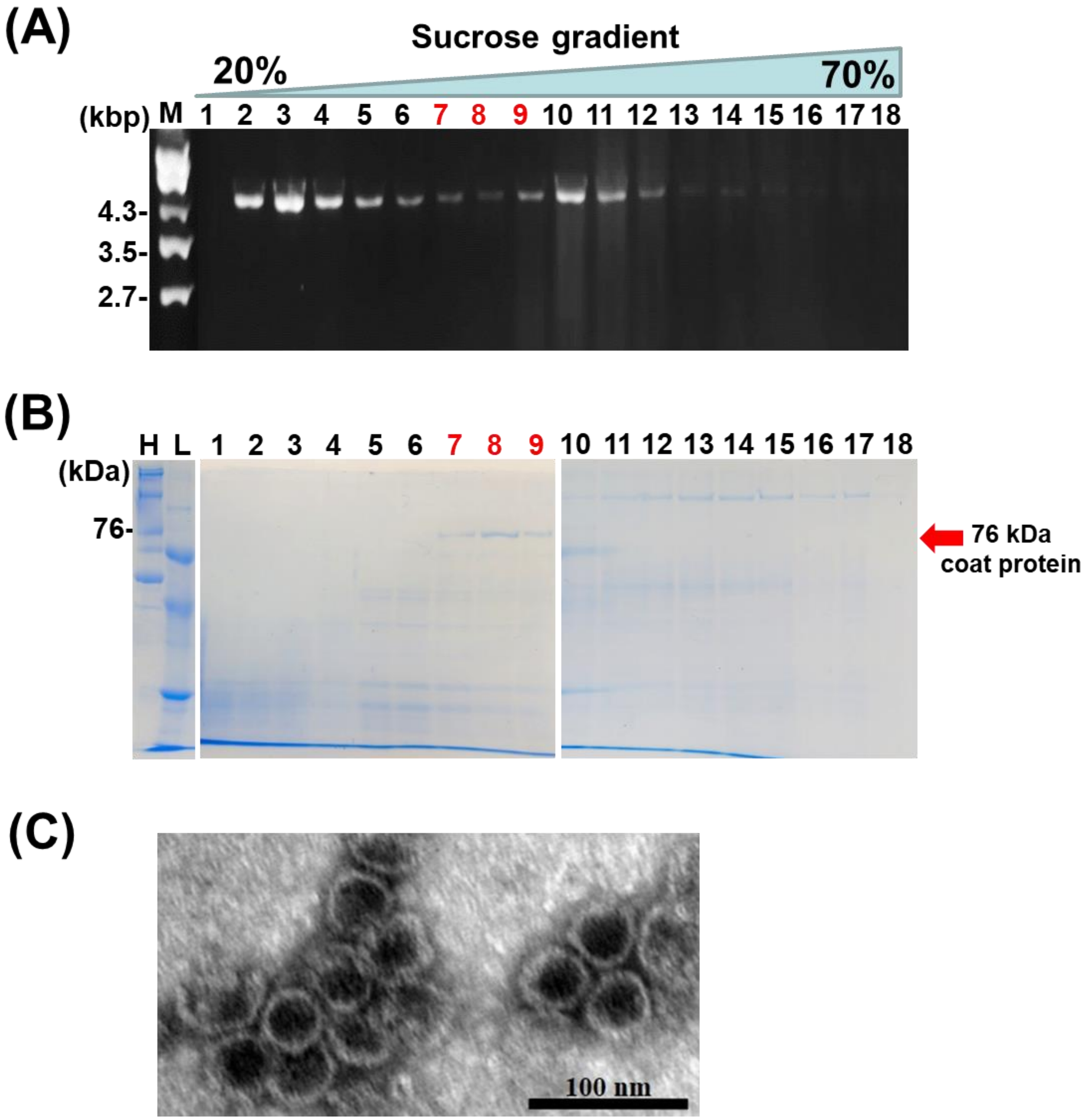

Supplemental Figure S6. ScV-L-A virus purification. The virus purification followed the published article (Powilleit et al., 2007), and resolved with 20-70% sucrose gradient, then fractioned into 18 fractions. The ScV-L-A viral particles **(C)** from fractions 7 to 9 (lanes 7-9), which showed 4.6 kb dsRNA genome **(A)** and 76 kDa coat protein **(B)**, were stained with 2% uranyl acetate and observed by TEM.

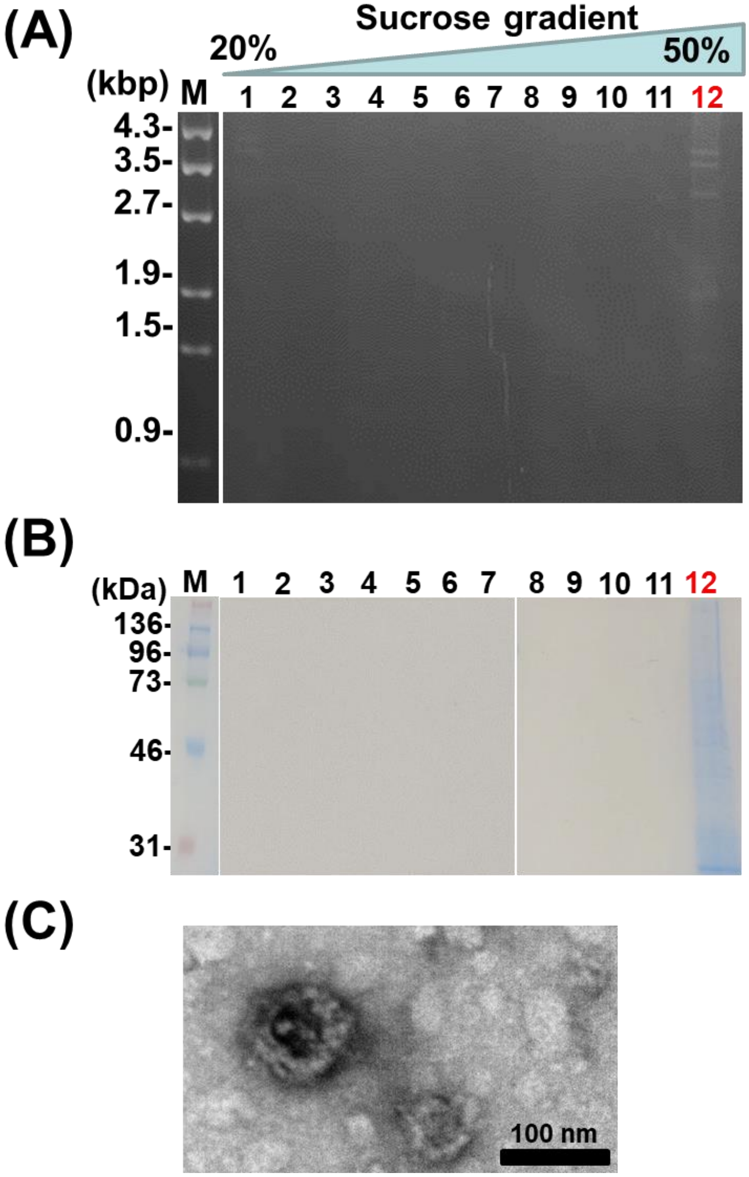

Supplemental Figure S7. MyRV1 virus purification. The virus purification followed the published article (Hillman et al., 2004), and resolved with 20-50% sucrose gradient, then fractioned into 12 fractions. The MyRV1 viral particles **(C)** from fractions 12 (lanes 12), which showed dsRNA genomes **(A)**, were stained with 2% uranyl acetate and observed by TEM. The specific protein bands were not obvious in the SDS-PAGE **(B)**.

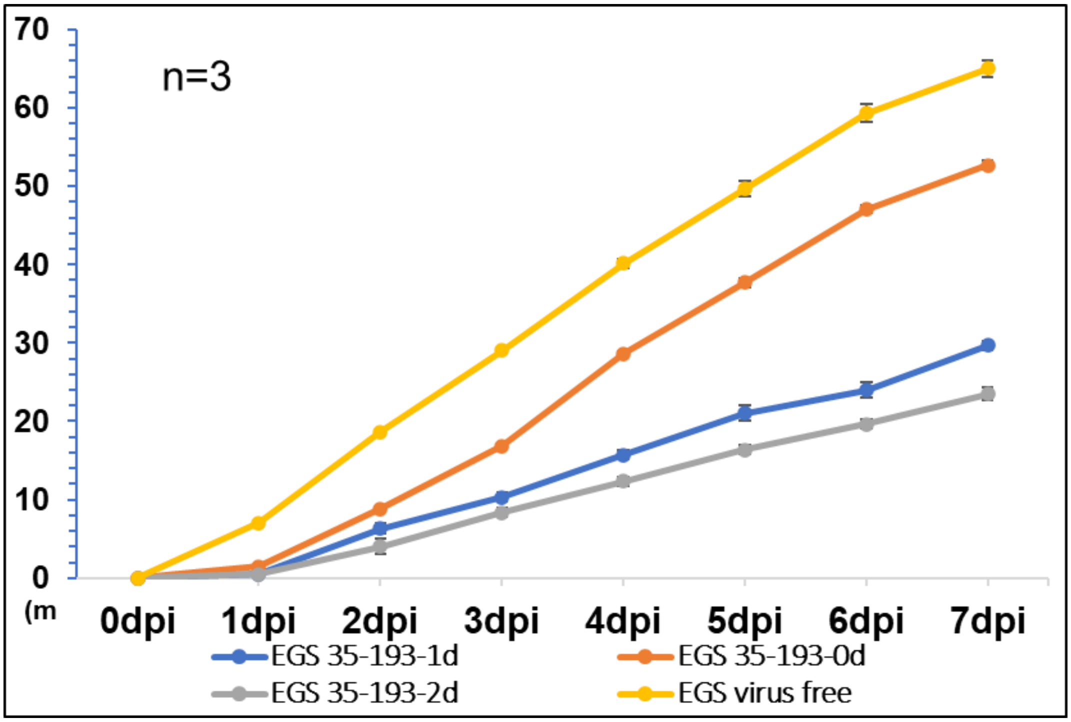

Supplemental Figure S8. Mycelial growth rates of AaV1-infected isolates and AaV1-free isolate on YGA plates (n = 3 replicates). Each isolate was cultured on YGA plates at 25 °C for 7 days, and the colony diameters were measured daily (excluding the agar plug with a diameter of 5mm).

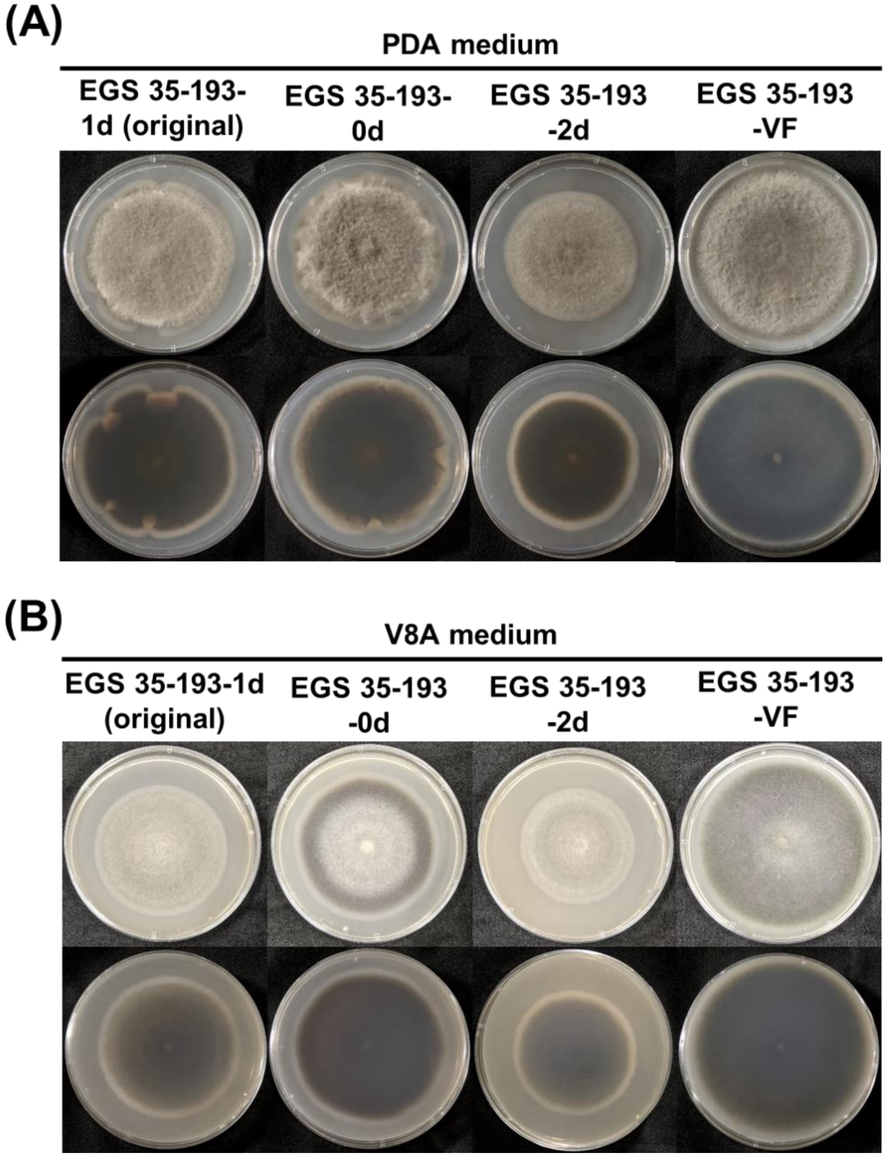

Supplemental Figure S9. Colony morphologies of AaV1-infected isolates and AaV1-free isolate on PDA and V8A media.

Supplemental Figure S10. Purified virus particles from AaV1-infected isolates (EGS 35-193-1d and EGS 35-193-2d). **(A)** EGS 35-193-1d **(B)** EGS35-193-2d. The purified 33nm virus particles were stained with 2% uranyl acetate and observed by TEM.

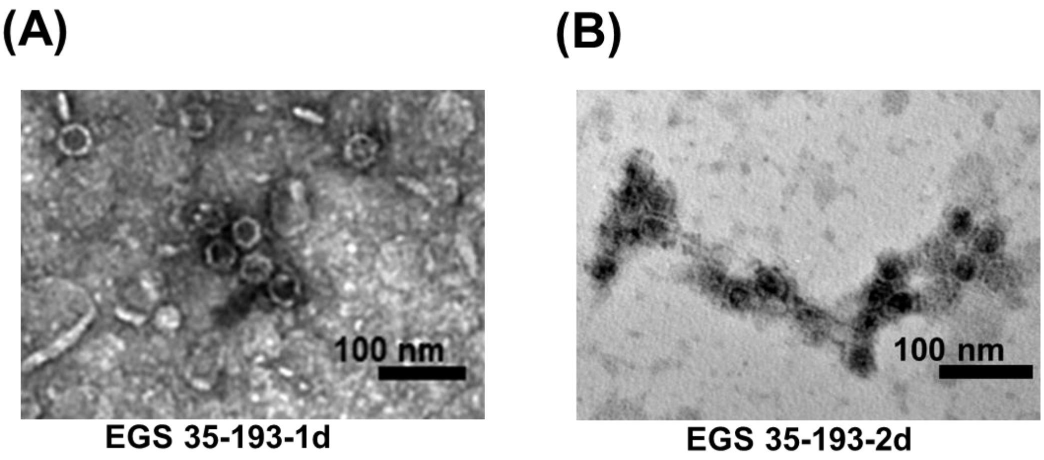

Supplemental Figure S10. Purified virus particles from AaV1-infected isolates (EGS 35-193-1d and EGS 35-193-2d). **(A)** EGS 35-193-1d **(B)** EGS35-193-2d. The purified 33nm virus particles were stained with 2% uranyl acetate and observed by TEM.

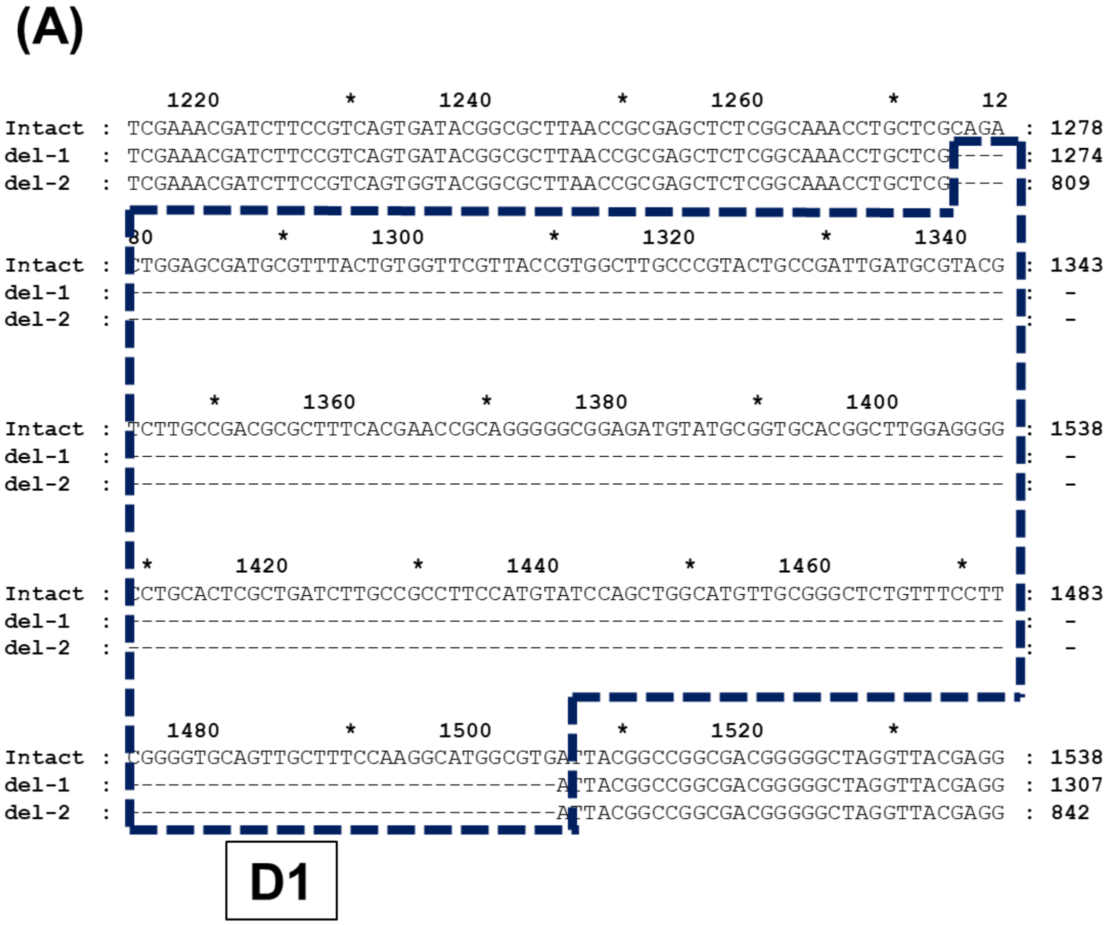
(A)
1220 * 1240 * 1260 * 12
Intact : TCGAAACGATCTTCCGTCAGTGATACGGCGCTTAACCGCGAGCTCTCGGCAAACCTGCTCGCAGA : 1278
del-1 : TCGAAACGATCTTCCGTCAGTGATACGGCGCTTAACCGCGAGCTCTCGGCAAACCTGCTCG---- : 1274
del-2 : TCGAAACGATCTTCCGTCAGTGGTACGGCGCTTAACCGCGAGCTCTCGGCAAACCTGCTCG---- : 809
80 * 1300 * 1320 * 1340
Intact : CTGGAGCGATGCGTTTACTGTGGTTCGTTACCGTGGCTTGCCCGTACTGCCGATTGATGCGTACG : 1343
del-1 : -- : -
del-2 : -- : -
* 1360 * 1380 * 1400
Intact : TCTTGCCGACGCGCTTTCACGAACCGCAGGGGGCGGAGATGTATGCGGTGCACGGCTTGGAGGGG : 1538
del-1 : -- : -
del-2 : -- : -
* 1420 * 1440 * 1460 *
Intact : CCTGCACTCGCTGATCTTGCCGCCTTCCATGTATCCAGCTGGCATGTTGCGGGCTCTGTTTCCTT : 1483
del-1 : -- : -
del-2 : -- : -
1480 * 1500 * 1520 *
Intact : CGGGGTGCAGTTGCTTTCCAAGGCATGGCGTGATTACGGCCGGCGACGGGGGCTAGGTTACGAGG : 1538
del-1 : --------------------------------ATTACGGCCGGCGACGGGGGCTAGGTTACGAGG : 1307
del-2 : --------------------------------ATTACGGCCGGCGACGGGGGCTAGGTTACGAGG : 842
D1

(B)

```
                  *        20         *        40         *        60         *
Intact:  CACAAAGCAATCGTATCGCCAGATACACATAAGTCTTTGACCTTGGTTCGTTATGTCTTCTGCTGTTTCT  :70
Del-1:   CACAAAGCAATCGTATCGCCAGATACACATAAGTCTTTGACCTTGGTTCGTTATGTCTTCTGCTGTTTCT  :70
Del-2:   CACAAAGCAATCGTATCGCCAGATACACATAAGTCTTTGACCTTGGTTCGTTATGTCTTCTGCTGTTTCT  :70

                80         *       100         *       120         *       140
Intact:  GATTTCTTTGTCTGCGGGGGGTTTAGTGAGGGGTGGTTGGAGTGCGATCTCCTTGATGAGCGTACCGCTT  :140
Del-1:   GATTTCTTTGTCTGCGGGGGGTTTAGTGAGGGGTGGTTGGAGTGCGATCTCCTTGATGAGCGTACCGCTT  :140
Del-2:   GATTTCTTTGTCTGCGGGGGGTTTAGTGAGGGGTGGTTG-------------------------------  :109

                  *       160         *       180         *       200         *
Intact:  TTCACCTTGCTGCGCACGTCGGGTCTGATGGCATGATCGATGTCCATCTTGAGAAAATTCCTGGCGTTCC  :210
Del-1:   TTCACCTTGCTGCGCACGTCGGGTCTGATGGCATGATCGATGTCCATCTTGAGAAAATTCCTGGCGTTCC  :210
Del-2:   ----------------------------------------------------------------------  : -

               220         *       240         *       260         *       280
Intact:  CGTTTCCCCATGGCATGTGGGGGGAGGGCTGCGTGATTTTGGTGGAGGCGGTCTCGGGGGCTTTGTTGAT  :280
Del-1:   CGTTTCCCCATGGCATGTGGGGGGAGGGCTGCGTGATTTTGGTGGAGGCGGTCTCGGGGGCTTTGTTGAT  :280
Del-2:   ----------------------------------------------------------------------  : -

                  *       300         *       320         *       340         *
Intact:  GTCGGTCTTCTCAAAAGGCCTGTTCAGCAGTGGGATGGGTTGGCTTGTGGCACTTGCCGTGTTGGTGATC  :350
Del-1:   GTCGGTCTTCTCAAAAGGCCTGTTCAGCAGTGGGATGGGTTGGCTTGTGGCACTTGCCGTGTTGGTGATC  :350
Del-2:   ----------------------------------------------------------------------  : -

               360         *       380         *       400         *       420
Intact:  TTGCTGGGATGTGCGAGGTGGTCGGTTGCGGCCGCCGTCGCGTTGGCATGCAGGTCGCTGAAACGGAGGT  :420
Del-1:   TTGCTGGGATGTGCGAGGTGGTCGGTTGCGGCCGCCGTCGCGTTGGCATGCAGGTCGCTGAAACGGAGGT  :420
Del-2:   ----------------------------------------------------------------------  :-

                  *       440         *       460         *       480         *
Intact:  GCTTGTCACAAGCTCCATAATGGTTGCGGTGCAGCGCGCGGCTATCGCGCTTGCTCCTGACGATATGGAG  :490
Del-1:   GCTTGTCACAAGCTCCATAATGGTTGCGGTGCAGCGCGCGGCCATCGCGCTTGCTCCTGACGATATGGAG  :490
Del-2:   ----------------------------------------------------------------------  : -

               500         *       520         *       540         *       560
Intact:  TTTTGGGACACCGTGCCTGTCCGTGTTTCTGCTGGAGGCTATACCTTCATCAAGGACATGTCCCTCGACT  :560
Del-1:   TTTTGGGACACCGTGCCTGTCCGTGTTTCTGCTGGAGGCTATACCTTCATCAAGGACATGTCCCTCGACT  :560
Del-2:   ----------------------------------------------------------------------  : -

                  *       580         *       600
Intact:  TGGCCGCGGAGTCAGAGGTTATTACCAGTGGTGCTGAGAA  :600
Del-1 :  TGGCCGCGGAGTCAGAGGTTATTACCAGTGGTGCTGAGAA  :600
Del-2 :  --------------GAGGTTATTACCAGTGGTGCTGAGAA  :135
```

D2

Supplemental Figure S11. In-frame deletion sites of AaV1 dsRNA2 genome. **(A)** D2 deletion site. From nt 1,275 to nt 1,505, length: 231 bp. **(B)** D2 deletion site. From nt 113 to nt 577, length: 465 bp.

(A)

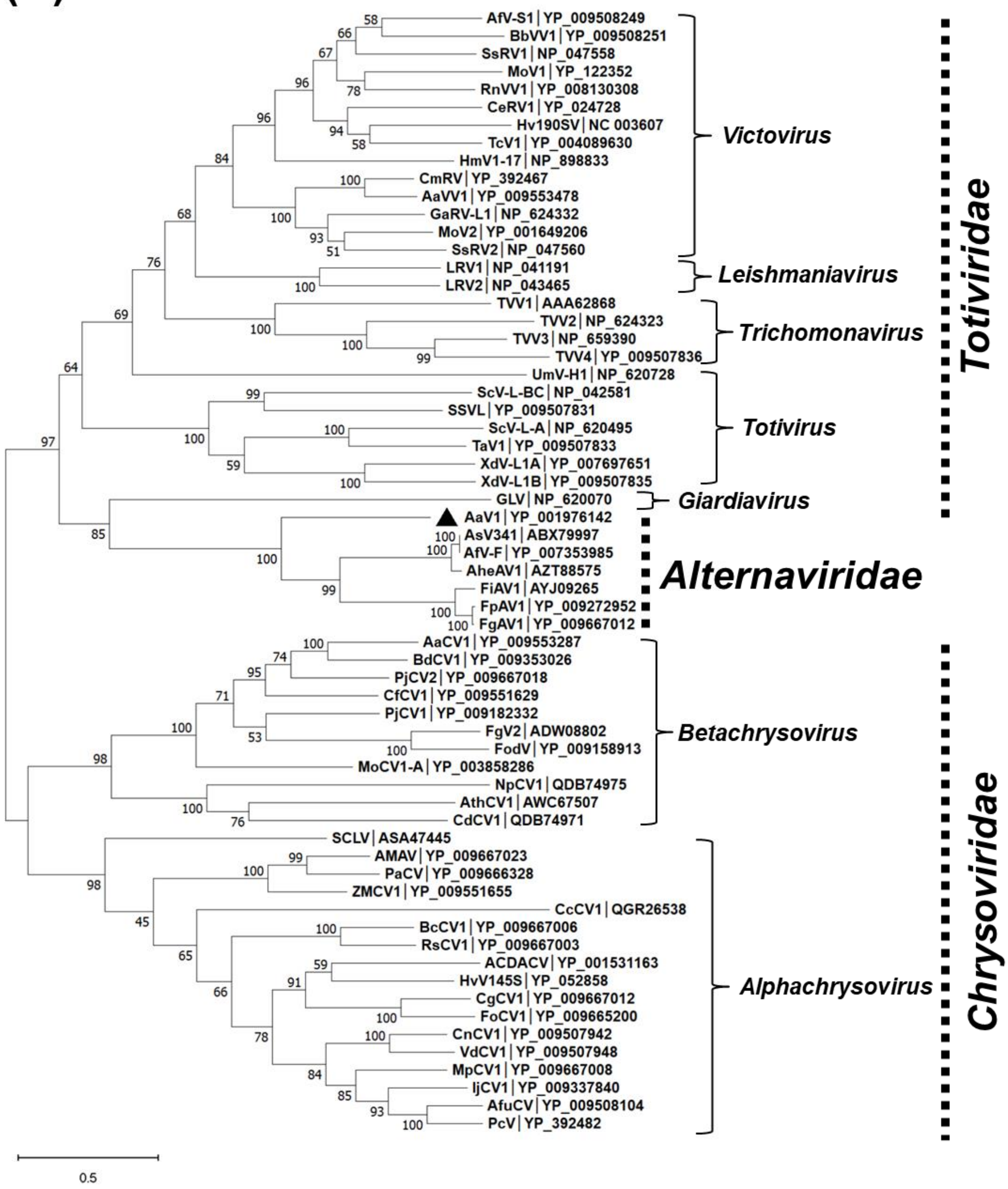
AfV-S1 | YP_009508249
BbVV1 | YP_009508251
SsRV1 | NP_047558
MoV1 | YP_122352
RnVV1 | YP_008130308
CeRV1 | YP_024728
Hv190SV | NC 003607
TcV1 | YP_004089630
HmV1-17 | NP_898833
CmRV | YP_392467
AaVV1 | YP_009553478
GaRV-L1 | NP_624332
MoV2 | YP_001649206
SsRV2 | NP_047560
LRV1 | NP_041191
LRV2 | NP_043465
TVV1 | AAA62868
TVV2 | NP_624323
TVV3 | NP_659390
TVV4 | YP_009507836
UmV-H1 | NP_620728
ScV-L-BC | NP_042581
SSVL | YP_009507831
ScV-L-A | NP_620495
TaV1 | YP_009507833
XdV-L1A | YP_007697651
XdV-L1B | YP_009507835
GLV | NP_620070
AaV1 | YP_001976142
AsV341 | ABX79997
AfV-F | YP_007353985
AheAV1 | AZT88575
FiAV1 | AYJ09265
FpAV1 | YP_009272952
FgAV1 | YP_009667012
AaCV1 | YP_009553287
BdCV1 | YP_009353026
PjCV2 | YP_009667018
CfCV1 | YP_009551629
PjCV1 | YP_009182332
FgV2 | ADW08802
FodV | YP_009158913
MoCV1-A | YP_003858286
NpCV1 | QDB74975
AthCV1 | AWC67507
CdCV1 | QDB74971
SCLV | ASA47445
AMAV | YP_009667023
PaCV | YP_009666328
ZMCV1 | YP_009551655
CcCV1 | QGR26538
BcCV1 | YP_009667006
RsCV1 | YP_009667003
ACDACV | YP_001531163
HvV145S | YP_052858
CgCV1 | YP_009667012
FoCV1 | YP_009665200
CnCV1 | YP_009507942
VdCV1 | YP_009507948
MpCV1 | YP_009667008
IjCV1 | YP_009337840
AfuCV | YP_009508104
PcV | YP_392482
Victovirus
Leishmaniavirus
Trichomonavirus
Totivirus
Giardiavirus
Totiviridae
Alternaviridae
Betachrysovirus
Alphachrysovirus
Chrysoviridae
0.5

(B)

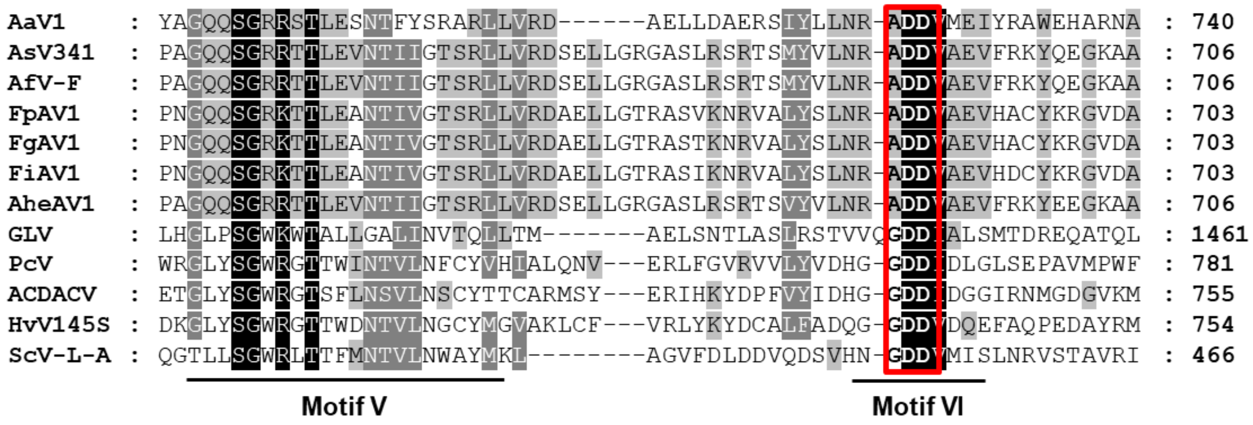

Supplemental Figure S12. (A) Phylogenetic analysis of *Alternaviridae*, *Totiviridae* and *Chrysoviridae* according to the amino acid sequences of RdRps. The amino sequences were retrieved from NCBI (Supplemental Table S2), then aligned using MUSCLE program. The phylogenetic tree was constructed using the Maximum Likelihood method and Poisson correction model by MEGA X (Kumar et al., 2018). The bootstrap values are marked next to the branches (100 replicates). **(B)** Multiple alignments of the RdRp motif VI of seven alternaviruses, three chrysoviruses, one totivirus, and one giardiavirus. Virus abbreviation: AaV1, Alternaria alternata virus 1; AsV341, Aspergillus mycovirus 341; AfV-F, Aspergillus foetidus mycovirus; FpAV1, Fusarium poae alternavirus 1; FgAV1, Fusarium graminearum alternavirus 1; FiAV1, Fusarium incarnatum alternavirus 1; AheAV1, Aspergillus heteromorphus alternavirus 1; GLV, Giardia lamblia virus; PcV, Penicillium chrysogenum virus; ACDACV, Amasya cherry disease-associated chrysovirus; Hv145SV, Helminthosporium victoriae 145S virus; ScV-L-A, Saccharomyces cerevisiae virus L-A.
